# Contrast subgraphs allow comparing homogeneous and heterogeneous networks derived from omics data

**DOI:** 10.1093/gigascience/giad010

**Published:** 2023-02-28

**Authors:** Tommaso Lanciano, Aurora Savino, Francesca Porcu, Davide Cittaro, Francesco Bonchi, Paolo Provero

**Affiliations:** Sapienza University of Rome, Rome 00185, Italy; Department of Molecular Biotechnology and Health Sciences, Molecular Biotechnology Center, University of Turin, Turin 10126, Italy; Sapienza University of Rome, Rome 00185, Italy; Center for Omics Sciences, San Raffaele Scientific Institute IRCSS, Milan 20132, Italy; CENTAI Institute, Corso Inghilterra 3, Turin 10138, Italy; Center for Omics Sciences, San Raffaele Scientific Institute IRCSS, Milan 20132, Italy; Department of Neurosciences “Rita Levi Montalcini,” University of Turin, Turin 10126, Italy

**Keywords:** Contrast subgraphs, gene networks, coexpression networks, protein interaction networks

## Abstract

**Background:**

Biological networks are often used to describe the relationships between relevant entities, particularly genes and proteins, and are a powerful tool for functional genomics. Many important biological problems can be investigated by comparing biological networks between different conditions or networks obtained with different techniques.

**Findings:**

We show that contrast subgraphs, a recently introduced technique to identify the most important structural differences between 2 networks, provide a versatile tool for comparing gene and protein networks of diverse origin. We demonstrate the use of contrast subgraphs in the comparison of coexpression networks derived from different subtypes of breast cancer, coexpression networks derived from transcriptomic and proteomic data, and protein–protein interaction networks assayed in different cell lines.

**Conclusions:**

These examples demonstrate how contrast subgraphs can provide new insight in functional genomics by extracting the gene/protein modules whose connectivity is most altered between 2 conditions or experimental techniques.

Key Points

- Contrast subgraphs extract the most significant structural difference between 2 networks while preserving node identity awareness.
- They can be used to compare biological networks derived from high-throughput experimental assays.
- Contrast subgraphs extracted from the comparison of gene/protein networks provide new insight in functional genomics.

## Introduction

The development of high-throughput methods in the past few decades has revolutionized biology by allowing the investigation of living systems from a global point of view, thanks to the various omics technologies [1]. The huge amount of data thus produced presents new analytical challenges for their interpretation and the extraction of useful and actionable biological information.

An important approach to such analytical task proceeds through the generation, from the high-throughput data, of *biological networks* expressing various types of relationships between the biological entities that have been measured (see [2] for a general introduction and [3] for a recent review). In some cases, the results of high-throughput measurements can be directly interpreted as networks, as in the case of protein interaction networks. In other cases, a network structure is built as an analytical tool to facilitate the extraction of biological information, as in the case of coexpression networks in which edges are established between genes showing correlated expression profiles in transcriptomic or proteomic assays. Many analytical tools developed in the context of network science can then be applied to such networks to extract biological information and formulate mechanistic hypotheses.

In many cases of biological interest, the most important questions can be answered not by simply analyzing a single biological system but by comparing 2 such systems to extract their fundamental differences. For example, when studying a disease, it is necessary to compare the diseased status to the normal one or different types of disease to each other. Moreover, different omics techniques can produce complementary insights into biological systems, and the investigation of such differences can shed light on the biological features best represented by each technique. When the system of interest has been described in terms of a biological network, techniques for network comparisons become the main tool for these investigations.

The bulk of the methods for network comparison can be categorized into 2 main classes: methods for the structural comparison of networks and methods for network alignment. Methods in the former class aim to detect global differences between networks in terms of the features considered in network science, such as connectivity distribution, clustering coefficient, and assortativity, and do not explicitly identify the individual nodes responsible for such differences. Methods for network alignment are mostly used to identify homologous modules in networks of different origin and are thus conceived to find similarities, rather than differences, between networks.

Recently, Lanciano et al. [4] proposed the extraction of *contrast subgraphs* as a method to identify the most important structural differences between 2 networks sharing the same nodes. In essence, contrast subgraphs are sets of nodes whose induced subgraphs are densely connected in one network and sparsely in the other (mathematical definitions and algorithms are found in the Methods). Contrary to most methods for structural comparison, contrast subgraphs are characterized by node identity awareness (i.e., identify the individual nodes that are responsible for the major differences between the networks). Thus, contrast subgraphs can be applied to pairs of networks sharing the same nodes or for which a suitable node-mapping function is available (such as when considering biological networks whose nodes are genes or proteins). The method allows rich downstream analyses based on domain-specific knowledge on the nodes. For instance, applications in which contrast subgraphs have been employed are social media [5] and neuroscience [4].

Here we apply contrast subgraphs to several comparisons of biological networks derived from high-throughput data, and we demonstrate how meaningful and novel biological information can be extracted from such comparisons. In particular, with respect to existing methods [6], contrast subgraphs exhibit 2 important advantages. First, the same technique can be used to compare homogeneous networks (i.e., obtained from the same high-throughput assay applied to different systems, such as coexpression networks obtained from 2 different types of cancer) or heterogeneous ones (obtained from different assays, such as protein coexpression and messenger RNA [mRNA] coexpression networks). Second, the method produces a hierarchically organized list of differentially connected modules that can be interpreted as representing separate biological processes.

## Results

To demonstrate how contrast subgraphs are useful in extracting biological information from the comparison of biological networks, we discuss 3 concrete examples, where the technique is applied to homogeneous networks (coexpression networks and protein–protein interaction [PPI] networks from different biological conditions) or heterogeneous ones (coexpression networks derived from transcriptomic and proteomic data).

### Coexpression networks in 2 subtypes of breast cancer

Transcriptomic assays have revealed that breast cancer is, from the molecular point of view, a highly heterogeneous disease. The most commonly used transcriptomic-based classification of this disease includes 5 subtypes (luminal A, luminal B, HER2 enriched, basal-like, and normal-like), where luminal A and basal-like are considered, respectively, the least and most aggressive subtypes [7]. We used 2 large repositories of breast cancer gene expression data—namely, the TCGA (https://www.cancer.gov/tcga)[8] and METABRIC [9]—to build coexpression networks separately for tumors classified as basal-like and as luminal A. The coexpression networks were based on Spearman’s correlation coefficients and built following the procedure used by WGCNA [10] (see Methods). We then extracted the contrast subgraphs from the comparison of the 2 subtype-specific networks, separately for each dataset. This is an example of comparison of homogeneous networks (i.e., obtained from the same assay) in 2 different conditions.

**Figure 1:**
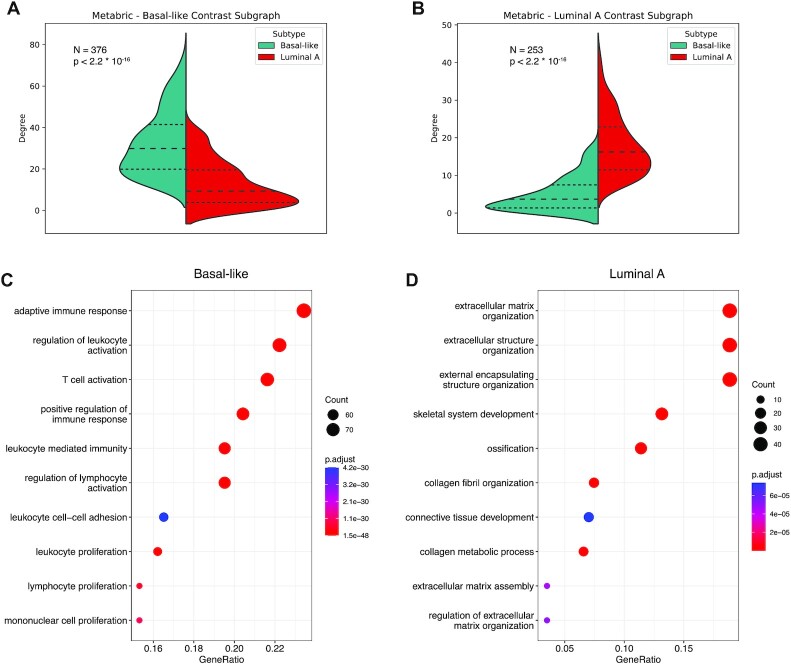
Contrast subgraphs between basal-like and luminal A subtypes for METABRIC. (A, B) Degree distribution of the nodes included in each contrast subgraph computed in the 2 coexpression networks. The *P*-value is obtained with the Mann–Whitney *U* test by comparing the 2 distributions. (C, D) Dotplots showing the enrichment of each contrast subgraph for Gene Ontology biological processes. The color gradient indicates the false discovery rate, while the dot size correlates with the number of nodes in the intersection between the contrast subgraph and the functional category. Only the top 10 most significant categories are shown. GeneRatio: fraction of genes in the gene set found in the contrast subgraph.

**Figure 2:**
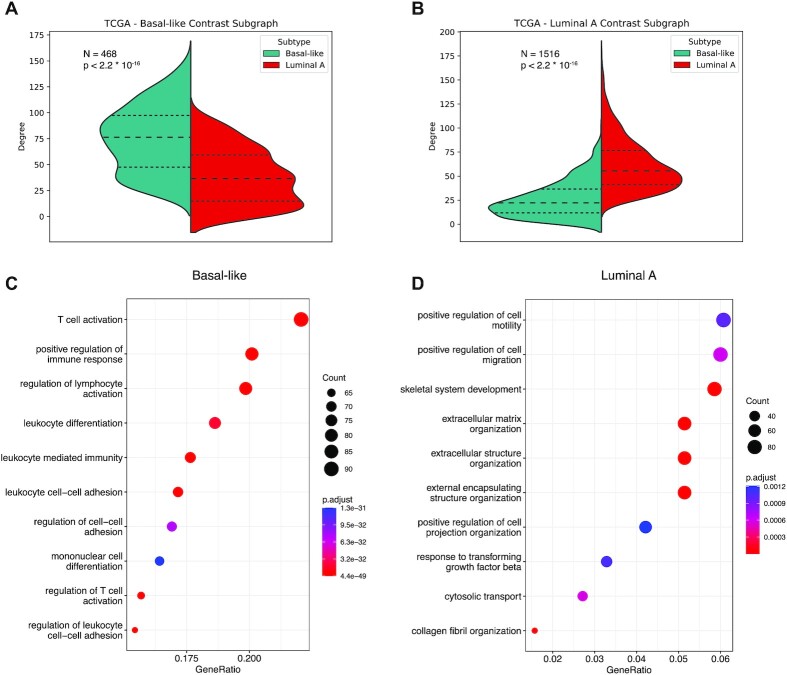
Same as Fig. 1 for the TCGA-based basal-like and luminal A coexpression networks.

Figs. 1 and 2 (A and B) represent the degree distribution for the first contrast subgraphs showing, as expected, a strong difference between the 2 subtypes (the genes in the first contrast subgraphs are listed in Supplementary Table S1). Analyzing these genes’ enrichment for functional categories, as annotated in the Gene Ontology (GO), we found immune-related processes to be coherently more coexpressed in the basal-like subtype, both in the TCGA and in the METABRIC cohort, while other processes related to tumor microenvironment, such as extracellular matrix organization, are more strongly coexpressed in the luminal A subtype (Figs. 1 and 2, panels C and D). This indicates that the tumor microenvironment, particularly immune cells and fibroblasts, play a prominent role in differentiating these 2 molecular subtypes. The full list of enriched GO categories is provided in Supplementary Table 2. Importantly, the results obtained with the 2 independent breast cancer cohorts show good agreement, with the top differential subgraphs significantly overlapping for both the basal-like and the luminal A subtypes in terms of both individual genes (all *P* < 2.2 · 10^−16^, Fisher test) and their functional enrichments (as shown in panels C and D of Figs. 1 and 2), supporting the reliability of the method.

**Table 1:** List of networks employed in this work

Dataset	|*V*|
Metabric	19,307
TCGA	16,995
Properseq	7,803
CPTAC	8,300

The coexpression networks discussed above were based on Spearman’s correlation. While this approach is the most commonly used, proportionality has been recently shown [11] to be a better alternative to correlation when building coexpression networks, allowing to avoid false positives due to the compositional nature of transcriptomic data. To verify whether our contrast subgraphs were robust with respect to the use of proportionality instead of correlation, we built proportionality-based networks (see Methods) and compared their contrast subgraphs to those obtained from correlation-based networks. The contrast subgraphs obtained with proportionality were in all cases highly similar to those obtained from correlation, with a Jaccard index >0.5 in all cases (0.71 for TCGA basal, 0.53 for TCGA luminal A, 0.79 for METABRIC basal, and 0.80 for METABRIC luminal A; all *P* = 2.2 · 10^−16^, exact Fisher test; Supplementary Table S3).

### Protein vs. mRNA coexpression in breast cancer

Coexpression networks are usually built, as we did above, from the results of transcriptomic assays, since these are less expensive than proteomic assays and thus available in large numbers. However, proteins, rather than mRNA molecules, are the predominant components of the molecular machinery performing cellular functions. Moreover, although transcriptomics studies commonly assume mRNA levels to be reliable indicators of corresponding protein levels, transcript and protein expression do not always correlate [12]. Indeed, synthesis and degradation rates of the 2 types of molecules can be substantially different [13]. Additionally, a wide range of posttranscriptional regulatory mechanisms, including translational repression by small noncoding RNAs and localization in processing bodies (P-bodies), could account for such discrepancies [14]. Therefore, it is reasonable to expect that coexpression networks built from protein abundance data could provide information that is complementary to that provided by mRNA-based coexpression networks and possibly more biologically relevant.

To analyze the differences between mRNA-based and protein-based coexpression networks, we used the proteomic data available from CPTAC [15] for a subset of the patients with breast cancer included in the TCGA and built a protein-based coexpression network, which was then compared using contrast subgraphs to the coexpression network obtained from the mRNA data of the same subset of patients. In this case, the networks to be compared are heterogeneous in that they are derived from 2 different assays.

**Figure 3:**
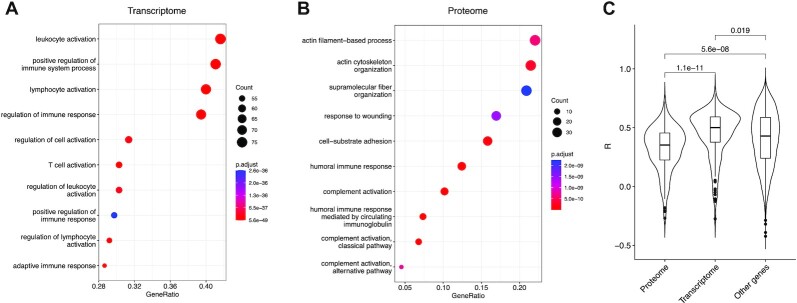
(A, B) Dotplots showing the enrichment of transcriptome vs. proteome contrast subgraphs for functional categories. The color gradient indicates false discovery rate, while dot size correlates with the number of nodes in the intersection between the contrast subgraph and the functional category. Only the top 10 most significant categories are shown. GeneRatio: fraction of genes in the gene set found in the contrast subgraph. (C) Violin plot showing Pearson’s correlation between transcriptomic and proteomic levels for genes in the top differential subgraph most connected at the proteome or transcriptome level, compared with genes not belonging to any of the 2 subgraphs.

The subgraphs with the strongest differential coexpression between the proteomic and transcriptomic data (listed in Supplementary Table S4) are enriched for immune categories. Of note, genes more connected at the protein level belong to categories such as “complement activation” and “regulation of humoral immune response,” while genes with functions in adaptive immunity are overrepresented among those with higher transcriptional coexpression (Fig. 3, full list in Supplementary Table S5). Moreover, the subgraph more connected at the protein level comprises genes with strikingly low correlation in their mRNA and protein expression (Fig. 3C), indicating that these genes are subject to additional regulatory layers, thus supporting their discrepant mRNA and protein coexpression (Cohen’s *d* for the difference in mRNA–protein correlation for genes in the transcriptome or proteome differential subgraphs: 0.52). This observation is in line with the complement cascade being mostly regulated through proteolytic activity and indicates that subgraphs more connected at the proteome level could better represent functional coupling of processes regulated at the posttranslational level.

### Protein interaction networks in human cell lines

The analysis of PPI networks can provide functional information complementary to that provided by transcriptomics. In particular, the comparison of such networks derived from different cell types or tissues can indicate those interactions that are specific to a biological context. We thus considered experimentally determined PPI networks in 3 human cell lines (HUVEC, HEK293T, and Jurkat) [16], derived from different human tissues: the vein of the umbilical cord (HUVEC), an embryonic human kidney (HEK293T), and T lymphocytes (Jurkat), so that we expect the respective PPI networks to reflect their diverse biological origins. These are widely used cell lines for which many different omics datasets have been produced by several labs and consortia. As the networks are derived from the same assay applied to different biological contexts, this is another example of a comparison of homogeneous networks. We extracted the contrast subgraphs for each of the 6 possible comparisons. Each contrast subgraph thus contains proteins with a higher density of interactions in one cell line compared with the other. The contrast subgraphs contained from a minimum of 143 (HEK vs. Jurkat and HEK vs. HUVEC) to a maximum of 204 (HUVEC vs. HEK) proteins.

It is important to verify that the contrast subgraphs thus extracted do not simply contain proteins that are differentially expressed when comparing the 3 cell lines. Indeed, we expect proteins that are upregulated in a cell line to be also easier to detect as interacting in the same cell line, without truly reflecting cell type–specific interactions. We compared the first contrast subgraphs obtained for each comparison with the list of upregulated genes obtained by comparing the transcriptomes of the same cell lines obtained by the Human Protein Atlas [17]. The proteins contained in the first contrast subgraph in HUVEC and, to a lesser extent, Jurkat cells did indeed significantly overlap the corresponding upregulated genes: for example, when comparing HUVEC to Jurkat cells, 112 proteins appearing in the contrast subgraph were also transcriptionally upregulated in HUVEC cells (expected by chance 30.5, *P* < 2.2 · 10^−16^, exact Fisher test). Such enrichment was not detected in HEK293T-specific contrast subgraphs. For example, the contrast subgraph obtained when comparing HEK293T to HUVEC cells contained 28 upregulated proteins (expected 20.5, *P* = 0.052). Therefore, we can be confident that the HEK293T-specific interactions contained in these contrast subgraphs are not exclusively due to transcriptional upregulation of the corresponding genes.

**Figure 4:**
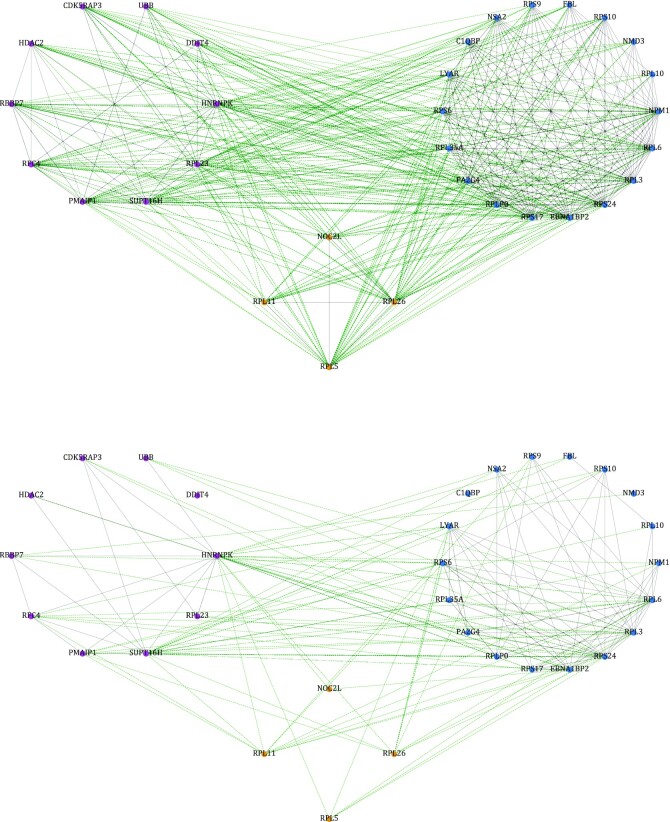
Subnetworks of the HEK293T (top) and HUVEC (bottom) cell line PPIs limited to proteins involved in “ribosome biogenesis” (blue), “signal transduction by p53 class mediator” (purple), and in both processes (yellow) together with their interactions. The green edges join a protein involved in one of the 2 biological processes to one involved in the other. The abundance of green edges in the top panel illustrates that the many interactions between these 2 processes are specific to the HEK293T cellular context. The figure for Jurkat cells would be identical, as all the interactions shown in the bottom panel happen to be common to the 3 cell lines.

We thus analyzed in more depth the 2 contrast subgraphs characterized by higher edge density in HEK293T compared with HUVEC and Jurkat cells, respectively. Remarkably, these 2 contrast subgraphs were identical and contained 143 proteins (Supplementary Table S6). Gene Ontology enrichment analysis of these proteins revealed 160 enriched biological processes (Supplementary Table S7), including many terms related to translation and ribosome biogenesis, on one hand, and many related to apoptosis and the TP53 pathway, on the other. Fig.   4 shows the proteins annotated “ribosome biogenesis” and “signal transduction by p53 class mediator” and their interactions in the HEK293T and HUVEC/Jurkat cell lines. These results suggest that HEK293T cells are particularly suitable for the investigation of the deep relationship between TP53 and the ribosome [ 18]. Indeed, these cells have been used in the original experimental investigation of this relationship [19].

These results show that contrast subgraphs can be used to identify cell type–specific modules of interacting proteins, thus facilitating the choice of the cells to be used for experimental assays.

## Methods

### Extraction of contrast subgraphs

Mining contrastive structures from networks has started recently to gain attention in the scientific literature. In this work, we leverage this recent literature to provide a first proposal of mining *contrast subgraphs* in the biological domain. Given 2 (potentially weighted) networks *A* = (*V*, *e*_*A*_(*V*)) and *B* = (*V*, *e*_*B*_(*V*)) defined over the same set of nodes *V*, we define a contrast subgraph as a set of nodes that is densely connected in one of the networks and sparse in the other. In order to quantify this property, different definitions of *contrast* have been provided in the literature.

Lanciano et al. [4] define the contrast subgraph as the set of nodes *S* ⊆*V* that maximizes the function $f(S) = e_A(S) - e_B(S) - \alpha \binom{|S|}{2}$, where *e*_*A*_(*S*) and *e*_*B*_(*S*) are the number of edges (or the sum of edges’ weight in case of a weighted network) in the subgraph induced by *S* in the networks *A* and *B*, respectively, and α is an input scalar. This definition aims at identifying a set of nodes, whose induced subgraph is dense in *A* and sparse in *B*. The regularization term $\alpha \binom{|S|}{2}$, governed by the parameter α, can be used to tune the target size of *S*: in fact, all the edges whose weight is smaller than α giving a negative contribution to the objective function, thus preventing larger solutions. To maximize this function, the authors map their problem to an instance of the Generalized Optimal Quasi Clique problem proposed by Cadena et al. [20]. Their algorithm is based on an Semi-Definite Programming optimization problem, which makes it practical only for smaller instances of networks (e.g., brain networks).

A variant formulation for this problem, by Yang et al. [21], aims at maximizing $f(S) = \frac{e_A(S) - e_B(S)}{|S|}$: they show that their problem is NP-hard and proposed a simple heuristic. It is worth observing that their problem corresponds to the classic densest subgraph problem (DSP) [22] on a weighted network, where the weight of an edge is given by *e*_*A*_(*S*) − *e*_*B*_(*S*). DSP is one of the most important primitives in graph mining, which has been studied extensively in literature for its many potential applications. Given a graph, it aims at finding the subgraph with maximum average degree (i.e., $\frac{e(S)}{|S|}$). When the graph is unweighted or positively weighted, DSP can be solved exactly in polynomial time by means of an inefficient max-flow-based algorithm [22]. An efficient $\frac{1}{2}$-approximation of the exact solution can be obtained by a greedy “peeling” algorithm that at every iteration removes the nodes with the current minimum degree and among all intermediate subgraphs produced by this process; in the end, it returns the one maximizing the objective function [23,24]. Unfortunately, when the graph has weights that can be negative, as in our case, these algorithmic results do not carry on (indeed, the contrast subgraph problem by Yang et al. [21] is NP-hard).

Tsourakakis et al. [5] recently analyzed the performance of the greedy peeling for the densest subgraph with negative weights problem. Let *deg*^+^(*v*) be the positive degree of node *v* (i.e., the sum of the weights of its positive edges) and *deg*^−^(*v*) its negative degree. They provide the following lower bound on the solution’s quality: $\frac{\rho ^{*}}{2} - \frac{\Delta }{2}$, where ρ* is the optimum value of the DSP problem, and $\Delta = \underset{v \in V}{\max }|deg^-(v)|$.

In order to improve such result, they propose a variant of the greedy peeling (algorithm 1), introducing a parameter *C* that governs the importance of *deg*^+^(*v*) in order to avoid the bad instances for which the greedy peeling could fail. It is sufficient to tune this parameter and run algorithm 1 several times to obtain a better result without a significant increase in computing time. Given its efficiency and scalability, and the fact that it has a certified lower bound on the quality of the solution provided, in our experiments,we employ algorithm 1 to mine the contrast subgraphs of coexpression and PPI networks.

In the literature reviewed above, the contrast subgraph is the one subgraph maximizing the contrastive objective function. However, although according to our algorithm 1, the extraction is limited to the subgraph that maximizes the contrast function, a straightforward heuristic to mine the top-*k* contrast subgraphs can be easily implemented by simply iterating algorithm 1 for *k* times, removing from the graph at each iteration the edges obtained in output.

**Figure:**
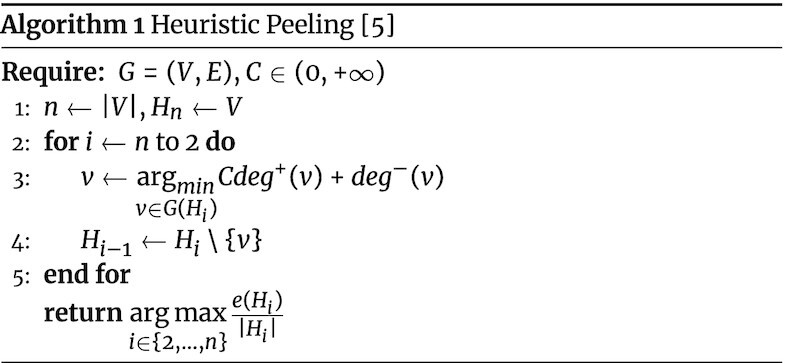


The algorithm takes as input a graph *G*, defined over the set of vertices *V* and the set of edges *E*, and a scalar *C* ∈ (0, +∞). It considers at the beginning the first candidate solution (*H*_*n*_) as the whole set of nodes *V* (line 1). Then, it selects iteratively the node *v* that has minimum global degree *Cdeg*^+^(*v*) + *deg*^−^(*v*) (line 3), removes it from the graph, and stores the current version of the graph (*H*_*i*_ at the *i*th iteration) as a candidate solution (line 4). Finally, it returns the solution among the candidate ones that maximizes the objective function $f(S) = \frac{e(S)}{|S|}$.

### Construction of coexpression networks

Normalized fragments per kilobase of transcript per million fragments mapped (FPKM) breast cancer data from the TCGA project and corresponding clinical annotations were obtained through TCGA biolinks [25], and METABRIC gene expression data and metadata were obtained from www.synapse.org (Synapse ID: syn1688369) [9]. All samples correspond to pretreatment primary breast tumors, analyzed in bulk without any cell-type separation, thus comprising not only cancer cells but also the tumor microenvironment. TCGA data acquisition and preprocessing were previously described [26,27]. No additional batch corrections or gene selection based on differential expression were performed. Probe names were converted in gene symbols, and for gene symbols corresponding to multiple probes, the most expressed probe across all samples was considered. ENSEMBL IDs were converted into gene symbols using biomaRt [28]. The TCGA data comprised 1,102 tumor samples (including 194 basal-like and 567 luminal A), while METABRIC comprised 1,981 tumor samples (including 328 basal-like and 719 luminal A). In TCGA, genes with FPKM <1 in more than 50 samples were filtered out, and data were log transformed using an offset of 1. Proteomic CPTAC data were downloaded from the original publication [15] and comprised 80 samples, including 77 in common with the TCGA transcriptomic dataset, which were used in the proteome vs. transcriptome network comparison. Adjacencies were computed using the Spearman’s correlation coefficient ρ between gene or protein expression, transformed into (0.5 · (1 + ρ))^12^. This transformation of the correlation value is identical to the one used by WGCNA [10] and provides a soft thresholding by suppressing small correlations without using a hard cutoff. Note that no hard cutoff was used, and the coexpression networks analyzed were all complete. Proportionality-based coexpression networks were built with the same procedure (including soft thresholding) by replacing the Spearman’s correlation coefficient with the ρ proportionality coefficient using the propr package [29]. Functional enrichment was performed using clusterProfiler [30], considering only categories with an adjusted *P* value less than 0.05. The overlap between contrast subgraphs obtained from different datasets was evaluated by Fisher’s exact test using as universe the genes present in both networks.

### Analysis of PPI constrast subgraphs

PPI networks obtained in HEK293T, HUVEC, and Jurkat cells were obtained from the supplementary material of the original publication [16]. PPI networks were filtered to include only proteins described in all cell lines. RNA sequencing data for the same cell lines were obtained form the Human Protein Atlas [17]. As these data do not contain replicates, lists of genes upregulated in each comparison were obtained by requiring |log_2_FC| > 1 on the normalized transcripts per million (TPM) values after logarithmic transformation with unit pseudocount.

## Discussion

Gene networks have proved to be a valuable tool to understand some general principles governing biological systems, revealing a modular organization of gene interactions [31], at least partly linked to shared function [32]. Comparing molecular networks across different contexts is nevertheless essential to explore the biology of dynamic systems, with gene and protein interactions changing over time or upon perturbations, such as disease or environmental stresses.

Many of the methods that have been developed for the comparison of biological network focus on structural properties and often lack node identity awareness; other methods focus on network alignment and aim at finding commonalities, rather than differences, between networks. Here we have shown that contrast subgraphs can provide a versatile tool to identify the modules with the strongest difference in connectivity between 2 networks. The method can be applied to networks of different biological and technical origin, and its node identity awareness allows downstream biological analyses, providing insight on the biological processes affected by differential connectivity. It should be noted that for the specific case of coexpression networks, several comparative methods that do retain node identity awareness have been developed (reviewed in [6,33]). However, most of these methods are specifically targeted to correlation-based networks and are not immediately applicable to other biological networks such as PPI networks. Other methods of network analysis that retain node identity awareness, the most prominent being probably community detection [34], cannot be directly applied to the task of finding the most significant differences between 2 networks. Our method could be considered a “supervised” version of community detection, where we maximize a function related to the difference in modularity, rather than modularity itself.

In this work, we applied contrast subgraphs to 3 pairs of biological networks to illustrate their usefulness, especially when followed by downstream functional enrichment analysis of the differential modules.

Breast cancer is one of the leading causes of mortality in women worldwide, for which no general efficacious treatment is available due to disease heterogeneity. It is usually classified according to gene expression profiling of the tumor (PAM 50 assay) into 5 molecular subtypes correlated with prognosis and response to treatments: luminal A, luminal B, basal-like, HER2 positive, and normal-like [35, 36]. In particular, the basal-like subtype does not respond to targeted treatments such as hormonal blockage or Herceptin and shows poor outcome.

We analyzed the different organization of c-expression networks between the aggressive basal-like and the relatively slowly growing luminal A subtypes. We find that the top differentially connected subgraphs comprise genes enriched for microenvironment-related functions in 2 independent cohorts with associated transcriptome datasets (METABRIC [9] and TCGA [8]). On one side, the subgraph with a stronger connection in the basal-like subtype is enriched for immune functions, while on the other side, the genes more connected in the luminal A subtype are enriched for categories such as “extracellular matrix,” indicative of microenvironmental regulation of structural components of the extracellular milieu. Indeed, tumor cells are surrounded by a varied ensemble of mutually interacting cell types, comprising immune cells, stromal cells, and blood vessels, among the most frequent cell types. These cells can either restrain tumor growth or support cancer cells by providing metabolites and growth factors and reshaping the extracellular matrix. Overall, nontumoral cells surrounding the tumor epithelium have been demonstrated to change their expression profiles [37] and to impact not only on tumor growth but also on disease progression and metastasis, as well as on drug resistance [10, 38, 39].

In particular, the immune system plays a fundamental role in cancer progression: at tumor onset, cytotoxic immune cells recognize and kill tumor cells, driving the evolution of less immunogenic cancer cells able to evade immune detection [40]. Paradoxically, immune cells such as anti-inflammatory M2 macrophages can have protumoral effects [41], and their distribution and composition change with tumorigenesis [37,42]. For these reasons, immune cells are currently being investigated as potential therapeutic targets [43, 44]. Interestingly, we previously reported that differentially coexpressed networks between normal and tumor tissues are often enriched for immune-related categories [33]. Specifically, the composition of the immune infiltrate has been shown to vary across breast cancer subtypes [45, 46], with higher T-cell infiltration in the most aggressive (i.e., basal-like) subtype [47], thus explaining the enrichment for “T-cell activation” of genes more strongly coexpressed in the basal-like subtype. On the other side, extracellular matrix remodeling can influence the stiffening of the collagen surrounding the tumor [48], influencing cancer cells’ migration and invasion [49]. Interestingly, both in the TCGA and in METABRIC, genes with stronger coexpression in the luminal A subtype are enriched for “extracellular matrix disassembly” (comprising matrix metalloproteases such as MMP2, MMP14, and MMP16), indicating that the activity of enzymes loosening the extracellular matrix fibrils is, as expected, higher in this subtype and thus confirming that the contrast subgraph method reliably retrieves robust and biologically informative sets of genes.

As a second application context, we employed our differential subgraph retrieval to compare biological networks derived from different kinds of molecular data: transcriptomics and proteomics. The possibility of comparing networks from different data types is indeed a strength of our method. We made use of the large breast cancer TCGA cohort of primary tumors, which have been profiled both through RNA sequencing and mass spectrometry [15], and defined gene modules whose connectivity can be revealed only at the proteomic level, likely due to posttranscriptional regulatory mechanisms influencing protein translation and degradation. Intriguingly, the 2 top differential modules show a significant difference in their transcript–protein agreement, supporting the hypothesis of intervening posttranscriptional mechanisms being involved in the proteome subgraph regulation. Again, these top differential subgraphs are enriched for immune-related categories. Interestingly, adaptive immune system genes are more connected at the transcriptional level, while innate immune system genes are more connected at the protein level.

Specifically, the proteomic subrgraph is significantly enriched for the complement cascade, comprising proteins such as C2, C3, C4A, C4B, and C5, in addition to complement regulators such as C4BPB. This could be interpreted as the innate immune system being poised for a rapid activation in the presence of a stimulus in the form of a pathogen or of a tumor cell, hence relying on a fast and coordinated translation of readily available transcripts or on the coordinated recruitment and degradation of constitutively produced proteins. Indeed, critical complement proteins (e.g., C3 and C5) are mainly produced by the liver and circulate in the serum [50], thus making varying levels of these proteins in breast independent of changes in gene expression *in situ*. Moreover, both activation and silencing of the complement system are mostly regulated at the protein level: a proteolytic cascade mediated by convertases leads to the amplification of complement activity [51, 52], while regulators such as CD55 and CD35 promote the degradation of C3 and C5 convertases, preventing the formation of the membrane attack complex [53]. The adaptive immune response, on the other hand, acts more slowly, requiring days or even weeks to become established. Therefore, transcription is not a limiting factor in its response, making transcriptionally regulated modules easily detectable. Many studies have found activation of the complement system in tumors and an increased complement activity in the sera of patients with cancer. In turn, this activation can lead to changes in adaptive immunity [54, 55], with the recruitment and activation of specific cell lymphocytic populations, which then reflect on gene expression. The CR2 complement receptor and the regulator of T-cell maturation PRDM1 [56] are indeed comprised in the transcriptomic subgraph and overall enriched for lymphocyte activation and differentiation.

In the third example, we showed that the comparison of PPI networks obtained from different human cell lines can reveal how proteins involved in different biological processes can have context-dependent interaction patterns. Importantly, such differences were not apparent from differential expression analysis of the same cell lines. These results suggest, in particular, that contrast subgraphs can be useful in selecting the cellular contexts most suitable for the experimental analysis of the interaction and mutual dependence of different biological processes.

An important limitation of our method, shared with community detection algorithms, is the lack of a quantitative measure of confidence on the contrast subgraphs obtained. This is partially compensated by the robustness shown by the method with respect to the use of independent datasets and of different methods for network construction, although we recognize that such extrinsic controls are not always available in practice.

## Conclusion

Contrast subgraphs are a promising and versatile method to identify the most relevant differences between biological networks while preserving node identity awareness, thus allowing the translation of such information into biological insight.

## Availability of Source Code and Requirements

- Project name: bio_cs
- Project homepage:  https://github.com/tlancian/bio_cs
- Operating system(s): Platform independent
- Programming language: Python
- Other requirements: none
- License: MIT
- biotoolsID: bio_contrast_subgraph
- RRID: SCR_022853

## Supplementary Material

giad010_GIGA-D-22-00204_Original_Submission

giad010_GIGA-D-22-00204_Revision_1

giad010_GIGA-D-22-00204_Revision_2

giad010_Response_to_Reviewer_Comments_Original_Submission

giad010_Response_to_Reviewer_Comments_Revision_1

giad010_Reviewer_1_Report_Original_Submission De-Shuang Huang -- 9/12/2022 Reviewed

giad010_Reviewer_1_Report_Revision_1 De-Shuang Huang -- 12/7/2022 Reviewed

giad010_Reviewer_2_Report_Original_Submission Raul Guantes -- 9/19/2022 Reviewed

giad010_Reviewer_2_Report_Revision_1 Raul Guantes -- 12/12/2022 Reviewed

giad010_Reviewer_3_Report_Original_Submission Thomas Schlitt -- 9/23/2022 Reviewed

giad010_Supplemental_Files

## Data Availability

The datasets supporting the results of this article and an archival copy of the code are available via the *GigaScience* database GigaDB [57].

## Additional Files

**Supplementary Table S1**. Genes in the first differential subgraphs comparing breast cancer molecular subtypes.

**Supplementary Table S2**. Gene Ontology categories significantly enriched in the corresponding differential subgraph comparing breast cancer molecular subtypes.

**Supplementary Table S3**. Contingency matrices for the overlap between the first differential subgraph in each comparison using gene expression correlation or proportionality as edge weight.

**Supplementary Table S4**. Genes in the first differential subgraphs comparing transcriptional and protein coexpression networks.

**Supplementary Table S5**. Gene Ontology categories significantly enriched in the corresponding differential subgraph comparing transcriptional and protein coexpression networks.

**Supplementary Table S6**. Genes in the first differential subgraph comparing HEK and Jurkat or HUVEC cell lines.

**Supplementary Table S7**. Gene Ontology categories significantly enriched in the differential subgraph comparing HEK and Jurkat or HUVEC cell lines.

## Abbreviations

DSP: densest subgraph problem; GO: Gene Ontology; mRNA: messenger RNA; PPI: protein–protein interaction.
