# Supplementary material for: Contrast subgraphs allow comparing homogeneous and heterogeneous networks derived from omics data
Source: Gigascience. 2023 Feb 28;12:giad010. doi: 10.1093/gigascience/giad010 (PMC9972522; doi:10.1093/gigascience/giad010)
Supplement: giad010_GIGA-D-22-00204_Revision_2 [file giad010_giga-d-22-00204_revision_2.pdf]

GigaScience

Contrast Subgraphs Allow Comparing Homogeneous and Heterogeneous Networks Derived from Omics Data

--Manuscript Draft--

**Manuscript Number:**	GIGA-D-22-00204R2
**Full Title:**	Contrast Subgraphs Allow Comparing Homogeneous and Heterogeneous Networks Derived from Omics Data
**Article Type:**	Technical Note
**Funding Information:**	
**Abstract:**	Background: Biological networks are often used to describe the relationships between relevant entities, in particular genes and proteins, and are a powerful tool for functional genomics. Many important biological problems can be investigated by comparing biological networks between different conditions, or networks obtained with different techniques.<br>Findings: We show that contrast subgraphs, a recently introduced technique to identify the most important structural differences between two networks, provide a versatile tool for comparing gene and protein networks of diverse origin. We demonstrate the use of contrast subgraphs in the comparison of coexpression networks derived from different subtypes of breast cancer; of coexpression networks derived from transcriptomic and proteomic data; and of protein-protein interaction networks assayed in different cell lines.<br>Conclusions: These examples demonstrate how contrast subgraphs can provide new insight in functional genomics by extracting the gene/protein modules whose connectivity is most altered between two conditions or experimental techniques.
**Corresponding Author:**	Paolo Provero<br>Università degli Studi di Torino<br>Torino, Torino ITALY
**Corresponding Author Secondary Information:**	
**Corresponding Author's Institution:**	Università degli Studi di Torino
**Corresponding Author's Secondary Institution:**	
**First Author:**	Tommaso Lanciano
**First Author Secondary Information:**	
**Order of Authors:**	Tommaso Lanciano
	Aurora Savino
	Francesca Porcu
	Davide Cittaro
	Francesco Bonchi
	Paolo Provero
**Order of Authors Secondary Information:**	
**Response to Reviewers:**	We have made all the changes required. Please note that the ORCID ids are included as comments in the "main.tex" file among the latex source files.
**Additional Information:**	
**Question**	**Response**
Are you submitting this manuscript to a special series or article collection?	No

Powered by Editorial Manager® and ProduXion Manager® from Aries Systems Corporation

**Experimental design and statistics**<br><br>Full details of the experimental design and statistical methods used should be given in the Methods section, as detailed in our Minimum Standards Reporting Checklist. Information essential to interpreting the data presented should be made available in the figure legends.<br><br>Have you included all the information requested in your manuscript?	Yes
**Resources**<br><br>A description of all resources used, including antibodies, cell lines, animals and software tools, with enough information to allow them to be uniquely identified, should be included in the Methods section. Authors are strongly encouraged to cite Research Resource Identifiers (RRIDs) for antibodies, model organisms and tools, where possible.<br><br>Have you included the information requested as detailed in our Minimum Standards Reporting Checklist?	Yes
**Availability of data and materials**<br><br>All datasets and code on which the conclusions of the paper rely must be either included in your submission or deposited in publicly available repositories (where available and ethically appropriate), referencing such data using a unique identifier in the references and in the "Availability of Data and Materials" section of your manuscript.<br><br>Have you have met the above requirement as detailed in our Minimum Standards Reporting Checklist?	Yes

Manuscript
Click here to access/download;Manuscript;BioContrastGigaScience.pdf

OXFORD (GIGA)^n SCIENCE

GigaScience, 2017, 1–10

doi: xx.xxxx/xxxx
Manuscript in Preparation
Paper

PAPER

Contrast Subgraphs Allow Comparing Homogeneous and Heterogeneous Networks Derived from Omics Data

Tommaso Lanciano[1,*], Aurora Savino[2,*], Francesca Porcu[1], Davide Cittaro[3], Francesco Bonchi[4,†] and Paolo Provero[3,5,†]

[1]Sapienza University of Rome, 00185 Rome, Italy and [2]Department of Molecular Biotechnology and Health Sciences, Molecular Biotechnology Center, University of Turin, 10126 Turin, Italy and [3]Center for Omics Sciences, San Raffaele Scientific Institute IRCSS, 20132 Milan, Italy and [4]CENTAI Institute, Corso Inghilterra 3, 10138 Turin, Italy and [5]Department of Neurosciences "Rita Levi Montalcini", University of Turin, 10126 Turin, Italy;

[*] Equal contributors
[†] Contact authors: bonchi@centai.eu; paolo.provero@unito.it

Abstract

Background: Biological networks are often used to describe the relationships between relevant entities, in particular genes and proteins, and are a powerful tool for functional genomics. Many important biological problems can be investigated by comparing biological networks between different conditions, or networks obtained with different techniques.
Findings: We show that contrast subgraphs, a recently introduced technique to identify the most important structural differences between two networks, provide a versatile tool for comparing gene and protein networks of diverse origin. We demonstrate the use of contrast subgraphs in the comparison of coexpression networks derived from different subtypes of breast cancer; of coexpression networks derived from transcriptomic and proteomic data; and of protein-protein interaction networks assayed in different cell lines.
Conclusions: These examples demonstrate how contrast subgraphs can provide new insight in functional genomics by extracting the gene/protein modules whose connectivity is most altered between two conditions or experimental techniques.

Key words: Contrast subgraphs; gene networks; coexpression networks; protein interaction networks.

Introduction

The development of high-throughput methods in the last few decades has revolutionized biology by allowing the investigation of living systems from a global point of view, thanks to the various omics technologies [1]. The huge amount of data thus produced present new analytical challenges for their interpretation and the extraction of useful and actionable biological information.

An important approach to such analytical task proceeds through the generation, from the high-throughput data, of *biological networks* expressing various types of relationships between the biological entities that have been measured (see [2] for a general introduction and [3] for a recent review). In some cases, the results of high-throughput measurements can be directly interpreted as networks, as in the case of protein interaction networks. In other cases, a network structure is built as an analytical tool to facilitate the extraction of biological information, as in the case of coexpression networks in which edges are established between genes showing correlated expression profiles in transcriptomic or proteomic assays. Many analytical tools developed in the context of network science can then be applied to such networks to extract biological information and formulate mechanistic hypotheses.

In many cases of biological interest, the most important questions can be answered not by simply analyzing a single biological system, but by comparing two such systems to extract their fundamental differences. For example, when studying a disease it is

Compiled on: February 4, 2023.
Draft manuscript prepared by the author.

Key Points

- Contrast subgraphs extract the most significant structural difference between two networks while preserving node identity awareness
- They can be used to compare biological networks derived from high-throughput experimental assays
- Contrast subgraphs extracted from the comparison of gene/protein networks provide new insight in functional genomics.

necessary to compare the diseased status to the normal one, or different types of disease to each other. Moreover, different omics techniques can produce complementary insights into biological systems, and the investigation of such differences can shed light on the biological features best represented by each technique. When the system of interest has been described in terms of a biological network, techniques for network comparisons become the main tool for these investigations.

The bulk of the methods for network comparison can be categorized into two main classes: methods for the structural comparison of networks and methods for network alignment. Methods in the former class aim to detect global differences between networks in terms of the features considered in network science, such as connectivity distribution, clustering coefficient, assortativity, etc., and do not explicitly identify the individual nodes responsible for such differences. Methods for network alignment are mostly used to identify homologous modules in networks of different origin, and are thus conceived to find similarities, rather than differences, between networks.

Recently, Lanciano et al. [4] proposed the extraction of *contrast subgraphs* as a method to identify the most important structural differences between two networks sharing the same nodes. In essence, contrast subgraphs are sets of nodes whose induced subgraphs are densely connected in one network and sparsely in the other (mathematical definitions and algorithms are found in the Methods). Contrary to most methods for structural comparison, contrast subgraphs are characterized by node identity awareness, *i.e.* identify the individual nodes that are responsible for the major differences between the networks. Thus, contrast subgraphs can be applied to pairs of networks sharing the same nodes, or for which a suitable node mapping function is available (such as when considering biological networks whose nodes are genes or proteins). The method allows rich downstream analyses based on domain-specific knowledge on the nodes. For instance, applications in which contrast subgraphs have been employed are social media [5] and neuroscience [4].

Here we apply contrast subgraphs to several comparisons of biological networks derived from high-throughput data, and we demonstrate how meaningful and novel biological information can be extracted from such comparisons. In particular, with respect to existing methods [6], contrast subgraphs exhibit two important advantages. First, the same technique can be used to compare homogeneous networks (that is, obtained from the same high-throughput assay applied to different systems, such as coexpression networks obtained from two different types of cancer) or heterogeneous ones (obtained from different assays, such as protein co-expression and mRNA co-expression networks). Second, the method produces a hierarchically organized list of differentially connected modules that can be interpreted as representing separate biological processes.

Results

To demonstrate how contrast subgraphs are useful in extracting biological information from the comparison of biological networks we discuss three concrete examples, where the technique is applied to homogeneous networks (coexpression networks and protein-protein interaction [PPI] networks from different biological conditions) or heterogeneous ones (coexpression networks derived from transcriptomic and proteomic data).

Coexpression networks in two subtypes of breast cancer

Transcriptomic assays have revealed that breast cancer is, from the molecular point of view, a highly heterogeneous disease. The most commonly used transcriptomic-based classification of this disease includes five subtypes (luminal-A, luminal-B, HER-2-enriched, basal-like, and normal-like), where luminal-A and basal-like are considered, respectively, the least and most aggressive subtypes [7]. We used two large repositories of breast cancer gene expression data, namely the TCGA (https://www.cancer.gov/tcga)[8] and METABRIC [9], to build coexpression networks separately for tumors classified as basal-like and as luminal-A. The coexpression networks were based on Spearman's correlation coefficients and built following the procedure used by WGCNA [10] (see Methods). We then extracted the contrast subgraphs from the comparison of the two subtype-specific networks, separately for each dataset. This is an example of comparison of homogeneous networks, i.e. obtained from the same assay, in two different conditions.

Figure 1 and Figure 2 (A and B) represent the degree distribution for the first contrast subgraphs showing, as expected, a strong difference between the two subtypes (the genes in the first contrast subgraphs are listed in Suppl. Table 1). Analysing these genes' enrichment for functional categories, as annotated in the Gene Ontology (GO), we found immune-related processes to be coherently more coexpressed in the basal-like subtype, both in the TCGA and in the METABRIC cohort, while other processes related to tumor microenvironment, such as extracellular matrix organization, are more strongly coexpressed in the luminal-A subtype (Figure 1 and 2, panels C and D). This indicates that the tumor microenvironment, and in particular immune cells and fibroblasts, play a prominent role in differentiating these two molecular subtypes. The full list of enriched GO categories is provided in Suppl. Table 2. Importantly, the results obtained with the two independent breast cancer cohorts show good agreement, with the top differential subgraphs significantly overlapping for both the basal-like and the luminal-A subtypes both in terms of individual genes (all $P < 2.2 \cdot 10^{-16}$, Fisher test), and their functional enrichments (as shown in panels C and D of Figs. 1 and 2) supporting the reliability of the method.

The coexpression networks discussed above were based on Spearman's correlation. While this approach the most commonly used, proportionality has been recently shown [11] to be a better alternative to correlation when building coexpression networks, allowing to avoid false positives due to the compositional nature of transcriptomic data. To verify whether our contrast subgraphs were robust with respect to the use of proportionality instead of correlation, we built proportionality-based networks (see Methods) and compared their contrast subgraphs to those obtained from correlation-based networks. The contrast subgraphs obtained with proportionality were in all cases highly similar to those obtained from correlation, with Jaccard index >0.5 in all cases (0.71 for TCGA basal, 0.53 for TCGA luminal-A, 0.79 for METABRIC basal, and 0.80 for METABRIC luminal-A; all P, $2.2 \cdot 10^{-16}$, exact Fisher test; Suppl.

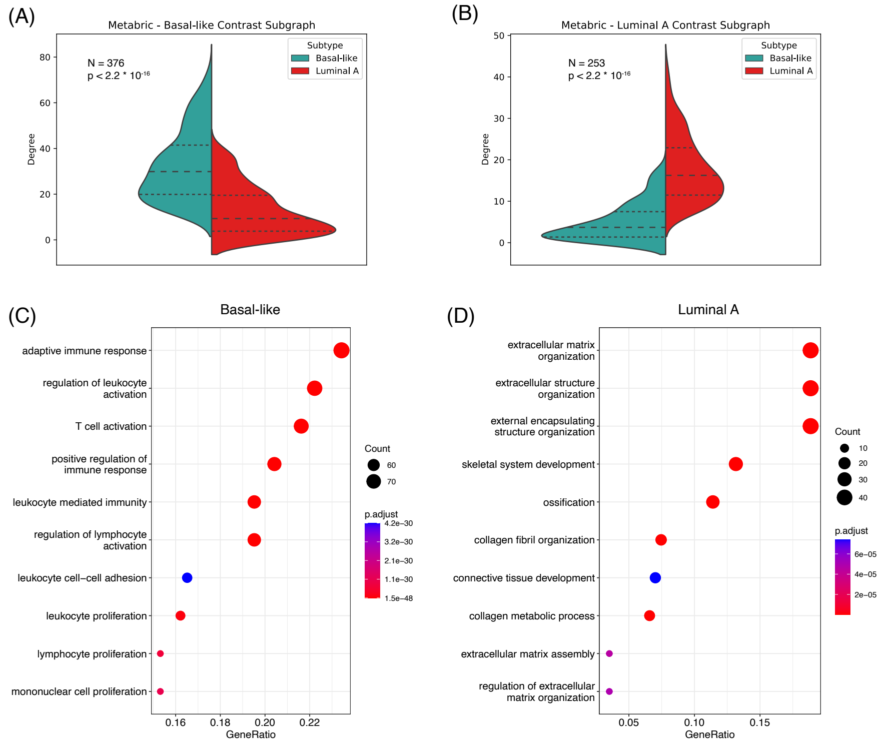

Figure 1. Contrast subgraphs between basal-like and luminal-A subtypes for METABRIC. A,B) Degree distribution of the nodes included in each contrast subgraph computed in the two coexpression networks. The P-value is obtained with the Mann-Whitney U test by comparing the two distributions. C,D) Dotplots showing the enrichment of each contrast subgraph for Gene Ontology biological processes. The colour gradient indicates the false discovery rate, while the dot size correlates with the number of nodes in the intersection between the contrast subgraph and the functional category. Only the top 10 most significant categories are shown. GeneRatio: fraction of genes in the geneset found in the contrast subgraph.

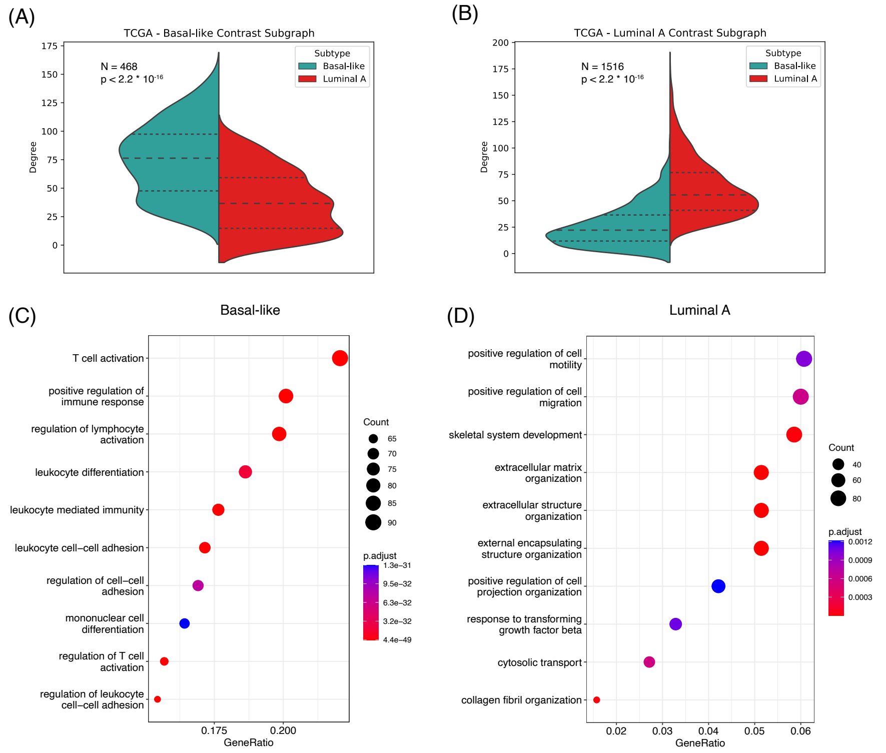

Figure 2. Same as Fig. 1 for the TCGA-based basal-like and luminal-A coexpression networks

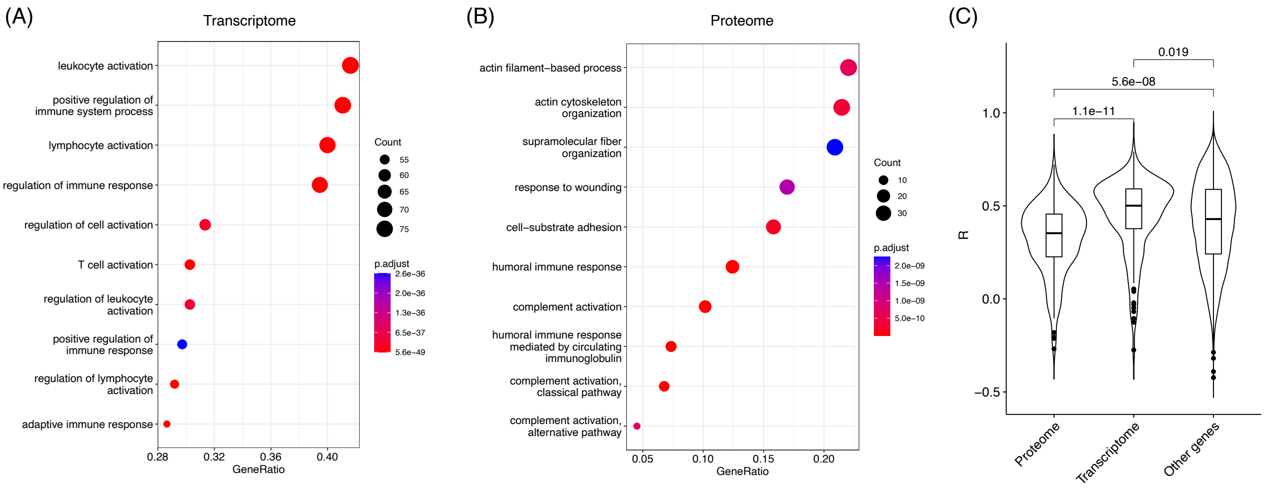

Figure 3. A, B) Dotplots showing the enrichment of transcriptome vs proteome contrast subgraphs for functional categories. The colour gradient indicates false discovery rate, while dot size correlates with the number of nodes in the intersection between the contrast subgraph and the functional category. Only the top 10 most significant categories are shown. GeneRatio: fraction of genes in the geneset found in the contrast subgraph. C) Violin plot showing Pearson's correlation between transcriptomic and proteomic levels for genes in the top differential subgraph most connected at the proteome or at the transcriptome level, compared with genes not belonging to any of the two subgraphs.

Table 3).

Protein vs mRNA coexpression in breast cancer

Coexpression networks are usually built, as we did above, from the results of transcriptomic assays, since these are less expensive than proteomic assays and thus available in large numbers. However, proteins, rather than mRNA molecules, are the predominant components of the molecular machinery performing cellular functions. Moreover, although transcriptomics studies commonly assume mRNA levels to be reliable indicators of corresponding protein levels, transcript and protein expression do not always correlate [12]. Indeed, synthesis and degradation rates of the two types of molecules can be substantially different [13]. Additionally, a wide range of post-transcriptional regulatory mechanisms, among which translational repression by small noncoding RNAs and localization in P bodies, could account for such discrepancies [14]. Therefore it is reasonable to expect that coexpression networks built from protein abundance data could provide information that is complementary to that provided by mRNA-based coexpression networks, and possibly more biologically relevant.

To analyze the differences between mRNA-based and protein-based coexpression networks we used the proteomic data available from CPTAC [15], for a subset of the breast cancer patients included in the TCGA, and built a protein-based coexpression network, which was then compared using contrast subgraphs to the coexpression network obtained from the mRNA data of the same subset of patients. In this case the networks to be compared are heterogeneous in that they are derived from two different assays.

The subgraphs with the strongest differential coexpression between the proteomic and transcriptomic data (listed in Suppl. Table 4) are enriched for immune categories. Of note, genes more connected at the protein level belong to categories such as "complement activation" and "regulation of humoral immune response", while genes with functions in the adaptive immunity are overrepresented among those with higher transcriptional coexpression (Figure 3, full list in Suppl. Table 5). Moreover, the subgraph more connected at the protein level comprises genes with strikingly low correlation in their mRNA and protein expression (Figure 3, C), indicating that these genes are subject to additional regulatory layers, thus supporting their discrepant mRNA and protein coexpression (Cohen's d for the difference in mRNA-protein correlation for genes in the transcriptome or proteome differential subgraphs: 0.52). This observation is in line with the complement cascade being mostly regulated through proteolytic activity, and indicates that subgraphs more connected at the proteome level could better represent functional coupling of processes regulated at the post-translational level.

Protein interaction networks in human cell lines

The analysis of PPI networks can provide functional information complementary to that provided by transcriptomics. In particular, the comparison of such networks derived from different cell types or tissues can indicate those interactions that are specific to a biological context. We thus considered experimentally determined PPI networks in three human cell lines (HUVEC, HEK293T, and JURKAT) [16], derived from different human tissues: the vein of the umbilical cord (HUVEC), an embryonic human kidney (HEK293T), and T lymphocytes (JURKAT), so that we expect the respective PPI networks to reflect their diverse biological origins. These are widely used cell lines for which many different omics datasets have been produced by several labs and consortia. As the networks are derived from the same assay applied to different biological contexts, this is another example of comparison of homogenous networks. We extracted the contrast subgraphs for each of the 6 possible comparisons. Each contrast subgraph thus contains proteins with higher density of interactions in one cell line compared with the other. The contrast subgraphs contained from a minimum of 143 (HEK vs JURKAT and HEK vs HUVEC) to a maximum of 204 (HUVEC vs HEK) proteins.

It is important to verify that the contrast subgraphs thus extracted do not simply contain proteins that are differentially expressed when comparing the three cell lines. Indeed, we expect proteins that are upregulated in a cell line to be also easier to detect as interacting in the same cell line, without truly reflecting cell type-specific interactions. We compared the first contrast subgraphs obtained for each comparison with the list of upregulated genes obtained by comparing the transcriptomes of the same cell lines obtained by the Human Protein Atlas [17]. The proteins contained in the first contrast subgraph in HUVEC and, to a lesser extent, JURKAT cells did indeed significantly overlap the corresponding upregulated genes: For example, when comparing HUVEC to JURKAT cells, 112 proteins appearing in the contrast subgraph were also transcriptionally upregulated in HUVEC cells (expected by chance 30.5, $P < 2.2 \cdot 10^{-16}$, exact Fisher test). Such enrichment was not

detected in HEK293T-specific contrast subgraphs. For example the contrast subgraph obtained when comparing HEK293T to HUVEC cells contained 28 upregulated proteins (expected 20.5, $P = 0.052$). Therefore, we can be confident that the HEK293T-specific interactions contained in these contrast subgraphs are not exclusively due to transcriptional upregulation of the corresponding genes.

We thus analyzed in more depth the two contrast subgraphs characterized by higher edge density in HEK293T compared with HUVEC and JURKAT cells, respectively. Remarkably, these two contrast subgraphs were identical, and contained 143 proteins (Suppl. Table 6). Gene Ontology enrichment analysis of these proteins revealed 160 enriched biological processes (Suppl. Table 7), including many terms related to translation and ribosome biogenesis on one hand, and many related to apoptosis and the TP53 pathway on the other. Figure 4 shows the proteins annotated "ribosome biogenesis" and "signal transduction by p53 class mediator" and their interactions in the HEK293T and HUVEC/JURKAT cell lines. These results suggest that HEK293T cells are particularly suitable for the investigation of the deep relationship between TP53 and the ribosome [18]. Indeed these cells have been used in the original experimental investigation of this relationship [19].

These results show that contrast subgraphs can be used to identify cell-type specific modules of interacting proteins, thus facilitating the choice of the cells to be used for experimental assays.

Methods

Extraction of contrast subgraphs

Mining contrastive structures from networks has started recently to gain attention in the scientific literature. In this work we leverage this recent literature to provide a first proposal of mining *contrast subgraphs* in the biological domain. Given two (potentially weighted) networks $A = (V, e_A(V))$ and $B = (V, e_B(V))$ defined over the same set of nodes V, we define a contrast subgraph as a set of nodes that is densely connected in one of the networks, and sparse in the other. In order to quantify this property, different definitions of *contrast* have been provided in the literature.

Lanciano et al. [4] define the contrast subgraph as the set of nodes $S \subseteq V$ that maximizes the function $f(S) = e_A(S) - e_B(S) - \alpha\binom{|S|}{2}$, where $e_A(S)$ and $e_B(S)$ are the number of edges (or the sum of edges' weight in case of a weighted network) in the subgraph induced by S in the networks A and B, respectively, and α is an input scalar. This definition aims at identifying a set of nodes, whose induced subgraph is dense in A and sparse in B. The regularization term $\alpha\binom{|S|}{2}$, governed by the parameter α, can be used to tune the target size of S: in fact, all the edges whose weight is smaller than α giving a negative contribution to the objective function, thus preventing larger solutions. To maximize this function, the authors map their problem to an instance of the Generalized Optimal Quasi Clique problem proposed by Cadena et al. [20]. Their algorithm is based on an Semi-Definite Programming optimization problem, that makes it practical only for smaller instances of networks, e.g. brain networks.

A variant formulation for this problem, by Yang et al. [21], aims at maximizing $f(S) = \frac{e_A(S)-e_B(S)}{|S|}$: they show that their problem is NP-hard and proposed a simple heuristic. It is worth observing that their problem corresponds to the classic *Densest Subgraph Problem* (DSP) [22] on a weighted network, where the weight of an edge is given by $e_A(S) - e_B(S)$. DSP is one the most important primitives in graph mining, that has been studied extensively in literature for its many potential applications. Given a graph, it aims at finding the subgraph with maximum average degree, i.e., $\frac{e(S)}{|S|}$. When the graph is unweighted or positively weighted, DSP can be solved exactly in polynomial time by means of an inefficient max-flow based algorithm [22]. An efficient $\frac{1}{2}$-approximation of the exact solution, can be obtained by a greedy "peeling" algorithm that at every iteration removes the nodes with the current minimum degree, and among all intermediate subgraphs produced by this process, in the end it returns the one maximizing the objective function [23, 24]. Unfortunately, when the graph has weights that can be negative, as in our case, these algorithmic results do not carry on (indeed, the contrast subgraph problem by Yang et al. [21] is NP-hard).

Tsourakakis et al. [5] recently analyzed the performance of the greedy peeling for the *Densest Subgraph with Negative Weights* (DSNW) problem. Let $deg^+(v)$ be the positive degree of node v, i.e., the sum of the weights of its positive edges, and $deg^-(v)$ its negative degree. They provide the following lower bound on the solution's quality: $\frac{\rho^*}{2} - \frac{\Delta}{2}$, where ρ^* is the optimum value of the DSP problem, and $\Delta = \max_{v \in V} |deg^-(v)|$.

In order to improve such result, they propose a variant of the greedy peeling (Algorithm 1), introducing a parameter C that governs the importance of $deg^+(v)$ in order to avoid the bad instances for which the greedy peeling could fail. It is sufficient to tune this parameter and run several times Algorithm 1 to obtain a better result without a significant increase in computing time. Given its efficiency and scalability, and the fact that it has a certified lower bound on the quality of the quality of the solution provided, in our experiments we employ Algorithm 1 to mine the contrast subgraphs of coexpression and PPI networks.

In the literature reviewed above, the contrast subgraph is the one subgraph maximizing the contrastive objective function. However, although according to our Algorithm 1 the extraction is limited to the subgraph that maximizes the contrast function, a straightforward heuristic to mine the top-k contrast subgraphs can be easily implemented, by simply iterating Algorithm 1 for k times, removing from the graph at each iteration the edges obtained in output.

Algorithm 1 Heuristic Peeling [5]

Require: $G = (V, E), C \in (0, +\infty)$
1: $n \leftarrow |V|, H_n \leftarrow V$
2: **for** $i \leftarrow n$ to 2 **do**
3: $\quad v \leftarrow \arg_{min}_{v \in G(H_i)} C deg^+(v) + deg^-(v)$
4: $\quad H_{i-1} \leftarrow H_i \setminus \{v\}$
5: **end for**
$\quad$ **return** $\arg\max_{i \in \{2,\ldots,n\}} \frac{e(H_i)}{|H_i|}$

The algorithm takes as input a graph G, defined over the set of vertices V and the set of edges E, and a scalar $C \in (0, +\infty)$. It considers at the beginning the first candidate solution (H_n) as the whole set of nodes V (line 1). Then, selects iteratively the node v that has minimum global degree $Cdeg^+(v) + deg^-(v)$ (line 3), removes it from the graph and stores the current version of the graph (H_i at the i-th iteration) as a candidate solution (line 4). Finally returns the solution among the candidate ones that maximizes the objective function $f(S) = \frac{e(S)}{|S|}$.

Construction of coexpression networks

Normalized (FPKM) breast cancer data from the TCGA project and corresponding clinical annotations were obtained through TCGA biolinks [25], and METABRIC gene expression data and metadata were obtained from www.synapse.org (Synapse ID: syn1688369) [9]. All samples correspond to pre-treatment primary breast tumors, analyzed in bulk without any cell-type separation, thus comprising not only cancer cells but also the tumor microenvironment. TCGA data acquisition and pre-processing were previously

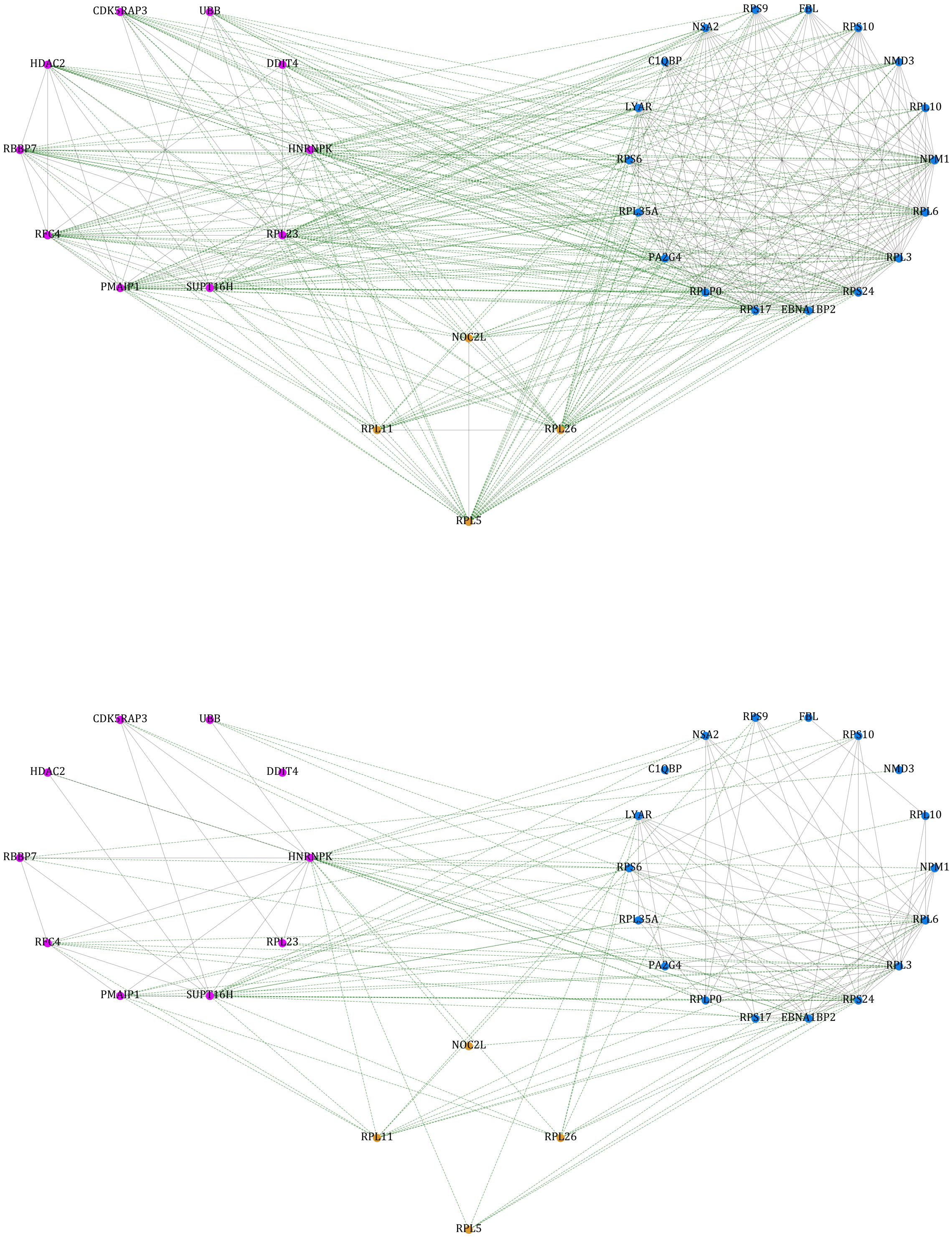

Figure 4. Subnetworks of the HEK293T (top) and HUVEC cells (bottom) PPIs limited to proteins involved in "ribosome biogenesis" (blue), "signal transduction by p53 class mediator" (purple) and in both processes (yellow) together with their interactions. The green edges join a protein involved in one of the two biological processes to one involved in the other. The abundance of green edges in the top panel illustrates that the many interactions between these two processes are specific of the HEK293T cellular context. The figure for JURKAT cells would be identical, as all the interactions shown in the bottom panel happen to be common to the three cell lines

Table 1. List of networks employed in this work.

Dataset	$\|V\|$
Metabric	19307
TCGA	16995
Properseq	7803
CPTAC	8300

described [26, 27]. No additional batch corrections nor gene selection based on differential expression were performed. Probe names were converted in Gene Symbols and, for gene symbols corresponding to multiple probes, the most expressed probe across all samples was considered. ENSEMBL IDs were converted into gene symbols using biomaRt [28]. The TCGA data comprised 1102 tumor samples (including 194 Basal-like and 567 Luminal A), while METABRIC comprised 1981 tumor samples (including 328 Basal-like and 719 Luminal A). In TCGA, genes with FPKM < 1 in more than 50 samples were filtered out, and data were log transformed using an offset of 1. Proteomic CPTAC data were downloaded from the original publication [15], and comprised 80 samples, including 77 in common with the TCGA transcriptomic dataset, which were used in the proteome vs transcriptome network comparison. Adjacencies were computed using the Spearman's correlation coefficient ρ between gene or protein expression, transformed into $(0.5 \cdot (1 + \rho))^{12}$. This transformation of the correlation value is identical to the one used by WGCNA [10], and provides a soft thresholding by suppressing small correlations without using a hard cutoff. Note that no hard cutoff was used, and the coexpression networks analyzed were all complete. Proportionality-based coexpression networks were built with the same procedure (including soft thresholding) by replacing the Spearman's correlation coefficient with the ρ proportionality coefficient using the propr package [29]. Functional enrichment was performed using clusterProfiler [30], considering only categories with an adjusted p-value less than 0.05. The overlap between contrast subgraphs obtained from different datasets was evaluated by Fisher's exact test using as universe the genes present in both networks.

Analysis of PPI constrast subgraphs

PPI networks obtained in HEK293T, HUVEC, and JURKAT cells were obtained from the Supplementary Material of the original publication [16]. PPI networks were filtered to include only proteins described in all cell lines. RNA-seq data for the same cell lines were obtained form the Human Protein Atlas [17]. As these data do not contain replicates, lists of genes upregulated in each comparison were obtained by requiring $|log_2 FC| > 1$ on the normalized TPM values after logarithmic transformation with unit pseudocount.

Discussion

Gene networks have proved to be a valuable tool to understand some general principles governing biological systems, revealing a modular organization of gene interactions [31], at least partly linked to shared function [32]. Comparing molecular networks across different contexts is nevertheless essential to explore the biology of dynamic systems, with gene and protein interactions changing over time or upon perturbations, such as disease or environmental stresses.

Many of the methods that have been developed for the comparison of biological network focus on structural properties and often lack node identity awareness; other methods focus on network alignment, and aim at finding commonalities, rather than differences, between networks. Here we have shown that contrast subgraphs can provide a versatile tool to identify the modules with the strongest difference in connectivity between two networks. The method can be applied to networks of different biological and technical origin, and its node identity awareness allows downstream biological analyses providing insight on the biological processes affected by differential connectivity. It should be noted that for the specific case of co-expression networks, several comparative methods that do retain node identity awareness have been developed (reviewed in [33, 6]). However most of these methods are specifically targeted to correlation-based networks and are not immediately applicable to other biological networks such as PPI networks. Other methods of network analysis that retain node identity awareness, the most prominent being probably community detection [34], cannot be directly applied to the task of finding the most significant differences between two networks. Our method could be considered as a "supervised" version of community detection, where we maximize a function related to the difference in modularity, rather than modularity itself.

In this work, we applied contrast subgraphs to three pairs of biological networks to illustrate their usefulness, especially when followed by downstream functional enrichment analysis of the differential modules.

Breast cancer is one of the leading causes of mortality in women worldwide, for which no general efficacious treatment is available due to disease heterogeneity. It is usually classified according to gene expression profiling of the tumor (PAM 50 assay) into five molecular subtypes correlated with prognosis and response to treatments: luminal-A, luminal-B, basal-like, HER2-positive and normal-like [35, 36]. In particular, the basal-like subtype does not respond to targeted treatments such as hormonal blockage or Herceptin, and shows poor outcome.

We analyzed the different organization of co-expression networks between the aggressive basal-like and the relatively slowly growing luminal-A subtypes. We find that the top differentially connected subgraphs comprise genes enriched for microenvironment-related functions in two independent cohorts with associated transcriptome datasets (METABRIC [9] and TCGA [8]). On one side, the subgraph with stronger connection in the basal-like subtype is enriched for immune functions, while, on the other side, the genes more connected in the luminal-A subtype are enriched for categories such as "Extracellular Matrix", indicative of microenvironmental regulation of structural components of the extracellular milieu. Indeed, tumor cells are surrounded by a varied ensemble of mutually-interacting cell types, comprising immune cells, stromal cells, and blood vessels, amongst the most frequent cell types. These cells can either restrain tumor growth or support cancer cells by providing metabolites and growth factors and reshaping the extracellular matrix. Overall, non-tumoral cells surrounding the tumor epithelium have been demonstrated to change their expression profiles [37] and to impact not only on tumor growth, but also on disease progression and metastasis, and on drug resistance [38, 39, 10].

In particular, the immune system plays a fundamental role in cancer progression: At tumor onset, cytotoxic immune cells recognize and kill tumor cells, driving the evolution of less immunogenic cancer cells able to evade immune detection [40]. Paradoxically, immune cells such as anti-inflammatory M2 macrophages can have pro-tumoral effects [41] and their distribution and composition changes with tumorigenesis [42, 37]. For these reasons, immune cells are currently being investigated as potential therapeutic targets [43, 44]. Interestingly, we previously reported that differentially coexpressed networks between normal and tumor tissues are often enriched for immune-related categories [33]. Specifically, the composition of the immune infiltrate has been shown to vary across breast cancer subtypes [45, 46], with higher T cell infiltration in the most aggressive (i.e. basal-like) subtype [47], thus explaining the enrichment for "T cell activation" of genes more strongly coexpressed in the basal-like subtype. On the other side, extracellular matrix remodelling can influence the stiffening of the collagen surrounding the tumor [48], influencing cancer cells' migration and invasion [49]. Interestingly, both in the TCGA and in METABRIC,

genes with stronger co-expression in the luminal-A subtype are enriched for "Extracellular matrix disassembly" (comprising matrix metalloproteases such as MMP2, MMP14 and MMP16), indicating that the activity of enzymes loosening the extracellular matrix fibrils is, as expected, higher in this subtype, and thus confirming that the contrast subgraph method reliably retrieves robust and biologically informative sets of genes.

As a second application context, we employed our differential subgraph retrieval to compare biological networks derived from different kinds of molecular data: transcriptomics and proteomics. The possibility of comparing networks from different data types is indeed a strength of our method. We made use of the large breast cancer TCGA cohort of primary tumors, which have been profiled both through RNA-seq and mass spectrometry [15], and defined gene modules whose connectivity can be revealed only at the proteomic level, likely due to post-transcriptional regulatory mechanisms influencing protein translation and degradation. Intriguingly, the two top differential modules show a significant difference in their transcript-protein agreement, supporting the hypothesis of intervening post-transcriptional mechanisms being involved in the proteome subgraph regulation. Again, these top differential subgraphs are enriched for immune-related categories. Interestingly, adaptive immune system genes are more connected at the transcriptional level, while innate immune system genes are more connected at the protein level.

Specifically, the proteomic subrgraph is significantly enriched for the complement cascade, comprising proteins such as C2, C3, C4A, C4B and C5, in addition to complement regulators such as C4BPB. This could be interpreted as the innate immune system being poised for a rapid activation in the presence of a stimulus in the form of a pathogen or of a tumor cell, hence relying on a fast and coordinated translation of readily available transcripts or on the coordinated recruitment and degradation of constitutively-produced proteins. Indeed, critical complement proteins (e.g. C3 and C5) are mainly produced by the liver and circulate in the serum [50], thus making varying levels of these proteins in breast independent on changes in gene expression in situ. Moreover, both activation and silencing of the complement system are mostly regulated at the protein level: a proteolytic cascade mediated by convertases leads to the amplification of complement activity ([51], [52]), while regulators such as CD55 and CD35 promote the degradation of C3 and C5 convertases, preventing the formation of the membrane attack complex (MAC) [53]. The adaptive immune response, on the other hand, acts more slowly, requiring days or even weeks to become established. Therefore, transcription is not a limiting factor in its response, making transcriptionally-regulated modules easily detectable. Many studies have found activation of the complement system in tumors and an increased complement activity in the sera of cancer patients. In turn, this activation can lead to changes in adaptive immunity [54, 55], with the recruitment and activation of specific cell lymphocytic populations, which then reflect on gene expression. The CR2 complement receptor and the regulator of T cell maturation PRDM1 [56] are indeed comprised in the transcriptomic subgraph, overall enriched for lymphocyte activation and differentiation.

In the third example we showed that the comparison of PPI networks obtained from different human cell lines can reveal how proteins involved in different biological processes can have context-dependent interaction patterns. Importantly, such differences were not apparent from differential expression analysis of the same cell lines. These results suggest, in particular, that contrast subgraphs can be useful in selecting the cellular contexts most suitable for the experimental analysis of the interaction and mutual dependence of different biological processes.

An important limitation of our method, shared with community detection algorithms, is the lack of a quantitative measure of confidence on the contrast subgraphs obtained. This is partially compensated by the robustness shown by the method with respect to the use of independent datasets and of different methods for network construction, although we recognize that such extrinsic controls are not always available in practice.

Conclusion

Contrast subgraphs are a promising and versatile method to identify the most relevant differences between biological networks while preserving node-identity awareness, thus allowing the translation of such information into biological insight.

Availability of source code and requirements

- Project name: bio_cs
- Project home page: https://github.com/tlancian/bio__cs
- Operating system(s): Platform independent
- Programming language: Python
- Other requirements: none
- License: MIT
- biotoolsID: bio_contrast_subgraph
- RRID: SCR_022853

Availability of supporting data and materials

The datasets supporting the results of this article and an archival copy of the code are available via the GigaScience database GigaDB [57].

Declarations

Ethical Approval (optional)

Not applicable.

Consent for publication

Not applicable.

Conflict of interest

The authors have no conflicts of interest related to this manuscript.

Author's Contributions

Project conception and coordination: FB, PP; software development: TL; data analysis and interpretation: TL, AS, FP, DC, PP, FB; manuscript writing: TL, AS, DC, PP, FB.

Acknowledgements

The results of this analysis are in whole or part based upon data generated by the TCGA Research Network: https://www.cancer.gov/tcga, accessed on 27 January 2021; and data generated by the Clinical Proteomic Tumor Analysis Consortium (NCI/NIH).

References

1. Huttenhower C, Hofmann O. A quick guide to large-scale genomic data mining. PLoS Computational Biology 2010 May;6(5):e1000779.

2. Barabási AL, Oltvai ZN. Network biology: understanding the cell's functional organization. Nature Reviews Genetics 2004 Feb;5(2):101–113. https://doi.org/10.1038/nrg1272.
3. Koutrouli M, Karatzas E, Paez-Espino D, Pavlopoulos GA. A guide to conquer the biological network era using graph theory. Frontiers in bioengineering and biotechnology 2020 Jan;8:34.
4. Lanciano T, Bonchi F, Gionis A. In: Explainable Classification of Brain Networks via Contrast Subgraphs New York, NY, USA: Association for Computing Machinery; 2020. p. 3308–3318. https://doi.org/10.1145/3394486.3403383.
5. Tsourakakis CE, Chen T, Kakimura N, Pachocki J. Novel Dense Subgraph Discovery Primitives: Risk Aversion and Exclusion Queries. In: Brefeld U, Fromont E, Hotho A, Knobbe A, Maathuis M, Robardet C, editors. Machine Learning and Knowledge Discovery in Databases Cham: Springer International Publishing; 2020. p. 378–394.
6. Bhuva DD, Cursons J, Smyth GK, Davis MJ. Differential co-expression-based detection of conditional relationships in transcriptional data: comparative analysis and application to breast cancer. Genome Biology 2019 Nov;20(1). https://doi.org/10.1186/s13059-019-1851-8.
7. Sørlie T, Perou CM, Tibshirani R, Aas T, Geisler S, Johnsen H, et al. Gene expression patterns of breast carcinomas distinguish tumor subclasses with clinical implications. Proceedings of the National Academy of Sciences of the United States of America 2001 Sep;98(19):10869–10874.
8. Network CGA. Comprehensive molecular portraits of human breast tumours. Nature 2012 Oct;490(7418):61–70.
9. Curtis C, Shah SP, Chin SF, Turashvili G, Rueda OM, Dunning MJ, et al. The genomic and transcriptomic architecture of 2,000 breast tumours reveals novel subgroups. Nature 2012 Apr;486(7403):346–352.
10. Zhang B, Horvath S. A general framework for weighted gene co-expression network analysis. Statistical Applications in Genetics and Molecular Biology 2005 Aug;4:Article17.
11. Lovell D, Pawlowsky-Glahn V, Egozcue JJ, Marguerat S, Bähler J. Proportionality: a valid alternative to correlation for relative data. PLoS computational biology 2015 March;11(3):e1004075. https://europepmc.org/articles/PMC4361748.
12. Liu Y, Beyer A, Aebersold R. On the Dependency of Cellular Protein Levels on mRNA Abundance. Cell 2016 Apr;165(3):535–550.
13. Vogel C, Marcotte EM. Insights into the regulation of protein abundance from proteomic and transcriptomic analyses. Nature Reviews Genetics 2012 Mar;13(4):227–232.
14. Wei YN, Hu HY, Xie GC, Fu N, Ning ZB, Zeng R, et al. Transcript and protein expression decoupling reveals RNA binding proteins and miRNAs as potential modulators of human aging. Genome Biology 2015 Feb;16(1). https://doi.org/10.1186/s13059-015-0608-2.
15. Mertins P, Mani DR, Ruggles KV, Gillette MA, Clauser KR, Wang P, et al. Proteogenomics connects somatic mutations to signalling in breast cancer. Nature 2016 Jun;534(7605):55–62.
16. Johnson KL, Qi Z, Yan Z, Wen X, Nguyen TC, Zaleta-Rivera K, et al. Revealing protein-protein interactions at the transcriptome scale by sequencing. Molecular Cell 2021 Oct;81(19):4091–4103.e9.
17. Uhlén M, Fagerberg L, Hallström BM, Lindskog C, Oksvold P, Mardinoglu A, et al. Proteomics. Tissue-based map of the human proteome. Science 2015 Jan;347(6220):1260419.
18. Deisenroth C, Franklin DA, Zhang Y. The Evolution of the Ribosomal Protein-MDM2-p53 Pathway. Cold Spring Harbor perspectives in medicine 2016 Dec;6(12).
19. Sloan KE, Bohnsack MT, Watkins NJ. The 5S RNP couples p53 homeostasis to ribosome biogenesis and nucleolar stress. Cell reports 2013 Oct;5(1):237–247.
20. Cadena J, Vullikanti AK, Aggarwal CC. On Dense Subgraphs in Signed Network Streams. In: 2016 IEEE 16th International Conference on Data Mining (ICDM); 2016. p. 51–60.
21. Yang Y, Chu L, Zhang Y, Wang Z, Pei J, Chen E. Mining Density Contrast Subgraphs. In: 2018 IEEE 34th International Conference on Data Engineering (ICDE) Los Alamitos, CA, USA: IEEE Computer Society; 2018. p. 221–232. https://doi.ieeecomputersociety.org/10.1109/ICDE.2018.00029.
22. Goldberg AV. Finding a Maximum Density Subgraph. USA; 1984.
23. Charikar M. Greedy Approximation Algorithms for Finding Dense Components in a Graph. In: Jansen K, Khuller S, editors. Approximation Algorithms for Combinatorial Optimization Berlin, Heidelberg: Springer Berlin Heidelberg; 2000. p. 84–95.
24. Asahiro Y, Iwama K, Tamaki H, Tokuyama T. Greedily Finding a Dense Subgraph. Journal of Algorithms 2000 Feb;34(2):203–221. Funding Information: 1Research supported in part by Science Research Grant 07458061, Ministry of Education, Japan. 2 Main part of the work done while author was at IBM Tokyo Research Laboratory.
25. Colaprico A, Silva TC, Olsen C, Garofano L, Cava C, Garolini D, et al. TCGAbiolinks: an R/Bioconductor package for integrative analysis of TCGA data. Nucleic Acids Research 2016 May;44(8):e71.
26. Ciriello G, Gatza ML, Beck AH, Wilkerson MD, Rhie SK, Pastore A, et al. Comprehensive Molecular Portraits of Invasive Lobular Breast Cancer. Cell 2015 Oct;163(2):506–519. https://doi.org/10.1016/j.cell.2015.09.033.
27. Comprehensive molecular portraits of human breast tumours. Nature 2012 Sep;490(7418):61–70. https://doi.org/10.1038/nature11412.
28. Durinck S, Spellman PT, Birney E, Huber W. Mapping identifiers for the integration of genomic datasets with the R/Bioconductor package biomaRt. Nature Protocols 2009 Jul;4(8):1184–1191.
29. Quinn TP, Richardson MF, Lovell D, Crowley TM. propr: An R-package for Identifying Proportionally Abundant Features Using Compositional Data Analysis. Scientific Reports 2017 Nov;7(1). https://doi.org/10.1038/s41598-017-16520-0.
30. Yu G, Wang LG, Han Y, He QY. clusterProfiler: an R package for comparing biological themes among gene clusters. Omics: a journal of integrative biology 2012 May;16(5):284–287.
31. Rives AW, Galitski T. Modular organization of cellular networks. Proceedings of the National Academy of Sciences 2003;100(3):1128–1133. https://www.pnas.org/doi/abs/10.1073/pnas.0237338100.
32. Eisen MB, Spellman PT, Brown PO, Botstein D. Cluster analysis and display of genome-wide expression patterns. Proceedings of the National Academy of Sciences of the United States of America 1998 Dec;95(25):14863–14868.
33. Savino A, Provero P, Poli V. Differential Co-Expression Analyses Allow the Identification of Critical Signalling Pathways Altered during Tumour Transformation and Progression. International Journal of Molecular Sciences 2020;21(24). https://www.mdpi.com/1422-0067/21/24/9461.
34. Javed MA, Younis MS, Latif S, Qadir J, Baig A. Community Detection in Networks: A Multidisciplinary Review. J Netw Comput Appl 2018 apr;108(C):87–111. https://doi.org/10.1016/j.jnca.2018.02.011.
35. Perou CM, Sørlie T, Eisen MB, van de Rijn M, Jeffrey SS, Rees CA, et al. Molecular portraits of human breast tumours. Nature 2000 Aug;406(6797):747–752. https://doi.org/10.1038/35021093.
36. Sørlie T, Perou CM, Tibshirani R, Aas T, Geisler S, Johnsen H, et al. Gene expression patterns of breast carcinomas distinguish tumor subclasses with clinical implications. Proceedings of the National Academy of Sciences 2001 Sep;98(19):10869–10874. https://doi.org/10.1073/pnas.191367098.
37. Savino A, De Marzo N, Provero P, Poli V. Meta-Analysis of Microdissected Breast Tumors Reveals Genes Regulated in

the Stroma but Hidden in Bulk Analysis. Cancers 2021;13(13). https://www.mdpi.com/2072-6694/13/13/3371.
38. Yang Y, Han L, Yuan Y, Li J, Hei N, Liang H. Gene co-expression network analysis reveals common system-level properties of prognostic genes across cancer types. Nature Communications 2014;5:3231.
39. Saelens W, Cannoodt R, Saeys Y. A comprehensive evaluation of module detection methods for gene expression data. Nature Communications 2018 Mar;9(1):1090.
40. Gonzalez H, Hagerling C, Werb Z. Roles of the immune system in cancer: from tumor initiation to metastatic progression. Genes &amp Development 2018 Oct;32(19-20):1267–1284. https://doi.org/10.1101/gad.314617.118.
41. Mantovani A, Marchesi F, Malesci A, Laghi L, Allavena P. Tumour-associated macrophages as treatment targets in oncology. Nature Reviews Clinical Oncology 2017 Jan;14(7):399–416. https://doi.org/10.1038/nrclinonc.2016.217.
42. Goff SL, Danforth DN. The Role of Immune Cells in Breast Tissue and Immunotherapy for the Treatment of Breast Cancer. Clinical Breast Cancer 2021;21(1):e63–e73. https://www.sciencedirect.com/science/article/pii/S1526820920301555.
43. Gun SY, Lee SWL, Sieow JL, Wong SC. Targeting immune cells for cancer therapy. Redox biology 2019 Jul;25:101174.
44. Dong Y, Wan Z, Gao X, Yang G, Liu L. Reprogramming Immune Cells for Enhanced Cancer Immunotherapy: Targets and Strategies. Frontiers in Immunology 2021;12. https://www.frontiersin.org/article/10.3389/fimmu.2021.609762.
45. Glajcar A, Szpor J, Hodorowicz-Zaniewska D, Tyrak KE, Okoń K. The composition of T cell infiltrates varies in primary invasive breast cancer of different molecular subtypes as well as according to tumor size and nodal status. Virchows Archiv 2019 Apr;475(1):13–23. https://doi.org/10.1007/s00428-019-02568-y.
46. Walens A, Olsson LT, Gao X, Hamilton AM, Kirk EL, Cohen SM, et al. Protein-based immune profiles of basal-like vs. luminal breast cancers. Laboratory Investigation 2021 Feb;101(6):785–793. https://doi.org/10.1038/s41374-020-00506-0.
47. Miyan M, Schmidt-Mende J, Kiessling R, Poschke I, de Boniface J. Differential tumor infiltration by T-cells characterizes intrinsic molecular subtypes in breast cancer. Journal of Translational Medicine 2016 Jul;14(1). https://doi.org/10.1186/s12967-016-0983-9.
48. Koorman T, Jansen KA, Khalil A, Haughton PD, Visser D, Rätze MAK, et al. Spatial collagen stiffening promotes collective breast cancer cell invasion by reinforcing extracellular matrix alignment. Oncogene 2022 Mar;41(17):2458–2469. https://doi.org/10.1038/s41388-022-02258-1.
49. Acerbi I, Cassereau L, Dean I, Shi Q, Au A, Park C, et al. Human breast cancer invasion and aggression correlates with ECM stiffening and immune cell infiltration. Integrative Biology 2015 May;7(10):1120–1134. https://doi.org/10.1039/c5ib00040h.
50. Merle NS, Noe R, Halbwachs-Mecarelli L, Fremeaux-Bacchi V, Roumenina LT. Complement System Part II: Role in Immunity. Frontiers in Immunology 2015 May;6. https://doi.org/10.3389/fimmu.2015.00257.
51. Roumenina LT, Daugan MV, Petitprez F, Sautès-Fridman C, Fridman WH. Context-dependent roles of complement in cancer. Nature Reviews Cancer 2019 Dec;19(12):698–715.
52. Hajishengallis G, Reis ES, Mastellos DC, Ricklin D, Lambris JD. Novel mechanisms and functions of complement. Nature Immunology 2017 Nov;18(12):1288–1298.
53. Killick J, Morisse G, Sieger D, Astier AL. Complement as a regulator of adaptive immunity. Seminars in Immunopathology 2017 Aug;40(1):37–48. https://doi.org/10.1007/s00281-017-0644-y.
54. Pio R, Corrales L, Lambris JD. The Role of Complement in Tumor Growth. In: Advances in Experimental Medicine and Biology Springer New York; 2013.p. 229–262. https://doi.org/10.1007/978-1-4614-5915-6_11.
55. Sasaki K, Takano S, Tomizawa S, Miyahara Y, Furukawa K, Takayashiki T, et al. C4b-binding protein -chain enhances antitumor immunity by facilitating the accumulation of tumor-infiltrating lymphocytes in the tumor microenvironment in pancreatic cancer. Journal of Experimental &amp Clinical Cancer Research 2021 Jun;40(1). https://doi.org/10.1186/s13046-021-02019-0.
56. Welsh RM. Blimp Hovers over T Cell Immunity. Immunity 2009 Aug;31(2):178–180. https://doi.org/10.1016/j.immuni.2009.08.005.
57. Tommaso L, Aurora S, Porcu Francesca, Davide C, Francesco B, Paolo P, Supporting data for "Contrast Subgraphs Allow Comparing Homogeneous and Heterogeneous Networks Derived from Omics Data". GigaScience Database; 2023. http://gigadb.org/dataset/102345.

Click here to access/download
Supplementary Material
main.tex

Click here to access/download
Supplementary Material
generic-color.tex

Click here to access/download
Supplementary Material
intro.tex

Click here to access/download
Supplementary Material
methods.tex

Click here to access/download
Supplementary Material
results.tex

Click here to access/download
Supplementary Material
supplementary.tex

Click here to access/download
Supplementary Material
conclusion.tex

Click here to access/download
Supplementary Material
discussion.tex

Click here to access/download
Supplementary Material
generic-color-brief.tex

Click here to access/download
Supplementary Material
biblio.bib

Click here to access/download
Supplementary Material
chato-notes.sty

Click here to access/download
Supplementary Material
generic.sty

Click here to access/download
Supplementary Material
ieeecolor.cls

Click here to access/download
Supplementary Material
main.bbl

Click here to access/download
Supplementary Material
oup-contemporary.cls

Click here to access/download
Supplementary Material
paper-refs.bib

Click here to access/download
Supplementary Material
vancouver-authoryear.bst

Click here to access/download
Supplementary Material
Figure 1.pdf

Click here to access/download
Supplementary Material
Figure 2.pdf

Click here to access/download
Supplementary Material
Figure 3.pdf

Click here to access/download
Supplementary Material
HEK_network.pdf

Click here to access/download
Supplementary Material
gigascience-logo.pdf

Click here to access/download
Supplementary Material
HUVEC_network.pdf
