# Supplementary figures and images for: Contrast subgraphs allow comparing homogeneous and heterogeneous networks derived from omics data
Source: Gigascience. 2023 Feb 28;12:giad010. doi: 10.1093/gigascience/giad010 (PMC9972522; doi:10.1093/gigascience/giad010)

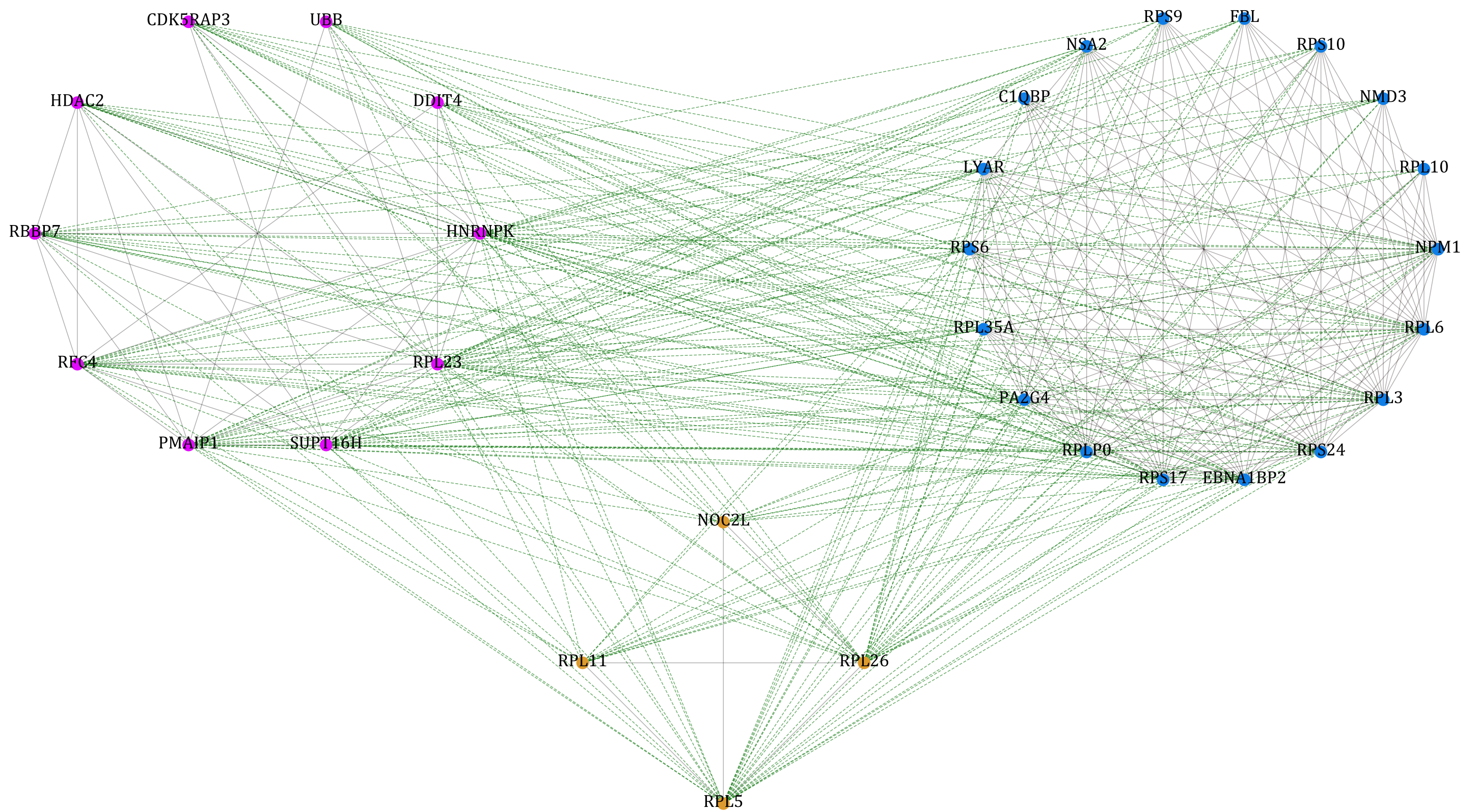

Supplement: giad010_Supplemental_Files [file giad010_supplemental_files.zip › HEK_network.pdf]

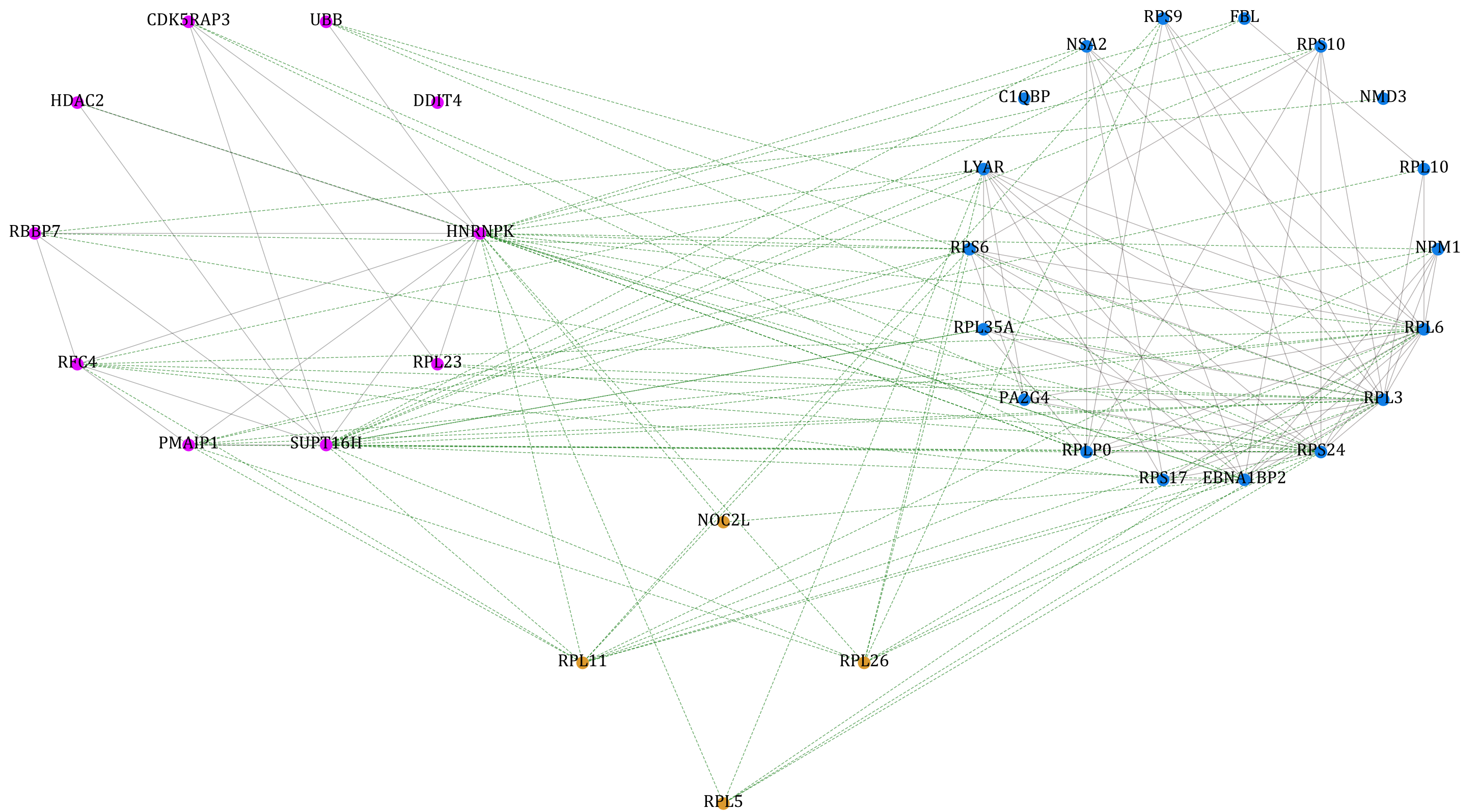

Supplement: giad010_Supplemental_Files [file giad010_supplemental_files.zip › HUVEC_network.pdf]
