# Supplementary material for: Contrast subgraphs allow comparing homogeneous and heterogeneous networks derived from omics data
Source: Gigascience. 2023 Feb 28;12:giad010. doi: 10.1093/gigascience/giad010 (PMC9972522; doi:10.1093/gigascience/giad010)
Supplement: giad010_Response_to_Reviewer_Comments_Original_Submission [file giad010_response_to_reviewer_comments_original_submission.pdf]

Author's Response To Reviewer Comments

Close

Editor

Please register any new software application in the bio.tools and SciCrunch.org databases to receive RRID (Research Resource Identification Initiative ID) and biotoolsID identifiers, and include these in your manuscript. This will facilitate tracking, reproducibility and re-use of your tool.

RESPONSE: We registered our software in both the repositories suggested. The identifiers are the following:

biotoolsID: bio_contrast_subgraph
RRID: SCR_022853

We included both in the manuscript (section “Availability of source code and requirements”)

===

Reviewer 1
Sub-graphs are generated by dividing the whole graph in a certain way, and the similarity and difference of the samples are described by the comparison between the sub-graphs. The authors should discuss the advantages of the proposed approach in a non-heuristically way compared with the previous methods. Besides that, I wonder why subgraphs need to be non-overlapping.

RESPONSE: We have added to the Discussion some considerations on the differences between our method and community detection algorithms, which represent another way of analyzing networks while preserving node-identity awareness, but cannot be directly applied to the task of finding the most significant differences between two networks. Our method could be considered as a "supervised" version of community detection where we maximize a function related to the difference in modularity, rather than modularity itself. However, we are not aware of other methods performing the same task as ours, that could be compared with our method in a quantitative way.
Regarding non-overlapping subgraphs, we thank the reviewer for the suggestion. Indeed, a simple adaptation of our heuristic can be implemented in which subgraphs do not need to be non-overlapping, by simply not considering at any iteration the edges (rather than the nodes) already obtained, leaving the nodes as part of the graph. We have changed the description of the method to reflect this improvement. Note that all the biological examples are unaffected by this change as we discuss only the first contrast subgraph.

====

For TCGA or other databases, I think the authors should state the details of the samples, such as the number of samples, sequencing technology, batch effects, etc. In addition, the authors should describe the relationship between the subgraphs and GO modules to explain the results and draw some biological conclusions.

RESPONSE: Details regarding the number of samples for the TCGA and METABRIC datasets have been included in the Methods, along with relevant citations of the original publications. No additional pre-processing steps, such as batch correction, were employed. The data from TCGA and METABRIC are never merged, but analyzed separately, and the final results in terms of GO enrichment are compared. Therefore, there is no need to correct for possible batch effects between the two datasets.

The biological interpretation of the results, especially in the light of the Gene Ontology enrichment, has been expanded in the discussion both for the comparison of Basal-like and Luminal A subtypes, and for the comparison of transcriptomic and proteomic networks.

====

The authors performed a similar analysis on protein networks and compared the results with RNA-seq, and get some conclusions. I'm a little confused whether the GO enrichment analysis of proteomics is to map the protein ID to the gene ID. If so, the authors can easily combine transcript co-expression and protein co-expression networks through ID-to-ID mapping, and I look forward to the results of such an analysis.

RESPONSE: We have clarified in the Introduction the fact that our method can be applied to pairs of graphs whose nodes are drawn from the same set (e.g. genes or proteins). Therefore, in the comparison between protein-level and gene-level coexpression, ID to ID mapping is used to translate the protein coexpression network into a gene coexpression network which is then compared with the gene coexpression network derived from RNA-sequencing data. Thus, we believe the analysis suggested by the reviewer is precisely the one that is presented in the section "Protein vs mRNA coexpression in breast cancer". In principle, it would also be interesting to integrate protein-level coexpression in the previous analysis of mRNA-level coexpression ("Coexpression networks in two subtypes of breast cancer"), but the limited number of samples for which proteomic data are available (80 samples in total, to be compared to > 1000 samples for each transcriptome dataset) would make such integration problematic.

====

I would like to know how the proposed method handles heterogeneous graphs by treating heterogeneous graphs as Homogeneous graph to generate subgraphs? I didn't figure out which dataset is the heterogeneous graph scenario.

RESPONSE: "Homogeneous" and "heterogeneous" refer to the experimental assays used to produce the data. So when comparing different RNA-based coexpression networks (section "Coexpression networks in two subtypes of breast cancer") or different protein-protein interaction networks (section "Protein interaction networks in human cell lines") we are comparing homogeneous networks, while in section "Protein vs mRNA coexpression in breast cancer" we are comparing heterogeneous networks (one derived from transcriptomics and one from proteomics). In some cases heterogeneous networks will need ID mapping prior to the extraction of contrast subgraphs, as discussed above, and in this sense we agree that they are treated as if they were homogeneous. We have clarified the distinction between homogeneous and heterogeneous in the Introduction and when discussing each example.

====

In addition to the elaboration of results such as degree and density differences between subgraphs, I would like to see the relationships between these results and the biological problems.

RESPONSE: Genes coding for metalloproteinases, complement cascade proteins and regulators of the complement system have now been included as specific examples in a wider discussion of the biological interpretations of our computational results, comparing the biological implications with previous knowledge on differences in the microenvironment composition across breast cancer subtypes and in the regulatory mechanisms of humoral and adaptive immunity.

====

Authors may consider citing the following articles on networks in molecular biology
Barabasi A L, Oltvai Z N. Network biology: understanding the cell's functional organization[J]. Nature reviews genetics, 2004, 5(2): 101-113.
Zhang, Q., He, Y., Wang, S., Chen, Z., Guo, Z., Cui, Z., ... & Huang, D. S. (2022). Base-resolution prediction of transcription factor binding signals by a deep learning framework[J]. PLoS computational biology, 2022, 18(3): e1009941.

Hu J X, Thomas C E, Brunak S. Network biology concepts in complex disease comorbidities[J]. Nature Reviews Genetics, 2016, 17(10): 615-629.
Z.-H. Guo, Z.-H. You, Y.-B. Wang, D.-S. Huang, H.-C. Yi, and Z.-H. Chen, "Bioentity2vec: Attribute-and behavior-driven representation for predicting multi-type relationships between bioentities." GigaScience 9.6 (2020): giaa032.
Z.-H. Guo, Z.-H. You, D.-S. Huang, H.-C. Yi, K. Zheng, Z.-H. Chen, Y.-B. Wang, MeSHHeading2vec: a new method for representing MeSH headings as vectors based on graph embedding algorithm[J]. Briefings in bioinformatics, 2021, 22(2): 2085-2095.

RESPONSE: We have added a citation to the Barabasi/Oltvai paper in the Introduction. The other suggested references seem to us too focused on specific types of biological networks to be useful here.

====

Reviewer 2
Of note, contrast subgraphs is not the only method that provides 'node identity awareness' when comparing networks. For instance, identification of network modules or community partitions are common methods to identify groups of nodes that highlight potentially relevant structural differences between two networks, and have been applied to many biological and other types of networks.

RESPONSE: We agree that community detection algorithms, in particular, do retain node identity awareness. However, to the best of our knowledge, they cannot be directly applied to the task of finding the most significant differences between two networks. Our method could be considered as a "supervised" version of community detection where we maximize a function related to the difference in modularity, rather than modularity itself. This point was added to the Discussion.

====

The discussion connecting their findings on structural differences between networks to potential biological functions is also a bit vague and could be worked out in more detail.

RESPONSE: According to the reviewer’s suggestion, we have now significantly expanded the biological discussion. In particular, genes coding for metalloproteinases, complement cascade proteins and regulators of the complement system have now been included as specific examples in a wider discussion of the biological interpretations of our computational results, comparing the biological implications with previous knowledge on differences in the microenvironment composition across breast cancer subtypes and in the regulatory mechanisms of humoral and adaptive immunity.

====

1 Coexpression networks for luminal and basal cancer subtypes:
a.- The authors don't give enough information about the data they are using to build these networks. How many samples/points are they using to calculate correlations? Do they correspond to different patients, expression dynamics after some treatment…? Is there any preprocessing in the data (e.g. differential expression with respect to healthy tissue) or they just take all quantified transcripts and proteins with minimal filtering (they only specified that filter out genes with FPKM < 1 in more than 50 samples in transcriptomic data)? How many nodes and links have the final coexpression networks?.

RESPONSE: Details specifying the number of samples for the TCGA and METABRIC datasets have now been included in the methods, along with relevant citations of the original publications. No additional pre-processing steps were employed. All the genes passing the filtering of FPKM>=1 in at least 50 samples have been included in the networks to obtain the most unbiased and complete representation of genes’ co-expressions, as suggested by the authors of the underlying network-building strategy (WGCNA, Ref. 10).

====

b. - To determine links between genes/proteins they calculate Spearman rho and transform it to

(0.5(1+rho)^12 to give a 'signed' network. But since Spearman correlation ranges between +1 and -1, this transformed quantity lies between 0 and 1, so I don't see the sign. Moreover, why the exponent 12 in the transformation??. Please clarify because I don't know if they are analyzing just weighted networks, unweighted networks or signed networks in the end because somehow they 'keep track' of the sign of rho. They spend some space in Methods discussing the extension of the contrast subgraph method to sign networks, but I don't know if they finally apply it, since coexpression networks built in this way and PPI networks are not signed.

RESPONSE: The networks here analyzed are complete weighted networks, generated using the same approach as the commonly employed WGCNA method (Ref. 10) for gene co-expression network construction. According to WGCNA nomenclature, in "unsigned" networks the absolute value of correlation is used to build the graph, while the "signed" networks employ the transformation (0.5(1+rho))^12 (with 12 being the default in WGCNA). In this case, negative correlations are close to zero, making strong connections represent positive correlations uniquely. Thus, clusters with high co-expression will contain genes with coherent gene expression across samples, without mixing them with genes having opposite gene expression trends. In this sense the network is "signed", as it keeps the sign into account. Both networks from transcriptomic and proteomic data were generated according to the same procedure. We have clarified in the text that we use the same procedure as WGCNA for the construction of the networks.

====

c. -Do they keep all links or use some cutoff in rho by magnitude/significance? Presumably yes, because otherwise the final network would be a clique and unmanageable, but they don't give any info on that. Again, which is the final size (node/links) of the coexpression networks?

RESPONSE: We do not employ any cut-off in the networks' pre-processing. The networks we analyze are complete weighted networks. Note however that the WGCNA transformation [½*(1 + rho)]^12 discussed above provides a form of "soft thresholding" by making all but the highest correlations close to zero. The graphs, despite the high number of edges, are manageable since the algorithm proposed runs in linear time, and there is no need to load the whole graph in memory. We added a table with the number of nodes for all the graphs.

=====

d.- As for coexpression networks based on relative abundance data as those from transcriptomic/proteomic experiments, it is well known that correlations may be misleading due to the possible large number of spurious correlations (see for instance Lovell at al., PLoS Computational Biology 11(3) (2015) e1004075). The use of correlations requires some justification, and at least to acknowledge the potential pitfalls of this measure.

RESPONSE: We used correlation-based coexpression networks built with the WGCNA recipe since this is the most commonly used method in the literature. As the reviewer points out, however, correlation-based networks are prone to false positives when used on compositional data, such as transcriptomics. Therefore, to check the robustness of our contrast subgraphs, we repeated the analysis of the breast cancer subtype-specific networks using proportionality, as suggested by Lovell et al., in place of correlation. Specifically, we built the networks with the same recipe used by WGCNA, including the soft thresholding, but replacing the Spearman correlation coefficient with the rho proportionality coefficient. Reassuringly, The contrast sub-
graphs obtained with proportionality were in all cases highly similar to those obtained from correlation, with Jaccard index >0.5 in all cases (0.71 for TCGA basal, 0.53 for TCGA luminal-A,
0.79 for METABRIC basal, and 0.80 for METABRIC luminal-A; all P, 2.2 · 10–16, exact Fisher test; Suppl. Table 3). These data have been discussed in the Results and included as Suppl. Table 3. Thus our method seems to be robust not only with respect to the use of different databases (TCGA or Metabric), but also with respect to the use of different measures of association to build the coexpression networks.

=====

e - How many nodes/links are in the first contrast subgraphs shown in Figures 1-2? Is the degree calculated within the whole network or just within the extracted subgraph?

RESPONSE: We now report the number of nodes of the contrast subgraphs in the figures. As the graphs are complete, the number of links are meaningless. The degree shown in the halved violin plots is defined as the sum of the weights of the edges.

=====

f - Page 4, last paragraph before 'Protein vs mRNA coexpression in breast cancer' section: 'the results obtained with the two independent breast cancer cohorts show good agreement, with the top differential subgraphs significantly overlapping for both the basal-like and the luminal-A subtypes (Fisher test p < 2.2 · 10-16)'. I guess the overlapping is in terms of functional annotations, how is this overlapping and the corresponding statistical test calculated?.

RESPONSE: The Fisher test refers to the overlap between the genes found in the contrast subgraphs generated with the two datasets, and was computed using as background all genes appearing in both networks, as we now specify in the Methods. The similarity of the functional enrichments can be appreciated from panels C and D of Figs. 1 and 2.

===

2.- Protein versus mRNA coexpression:

a. - Please provide again information about the number of samples, how the 'subset of breast cancer patients included in the TCGA' is chosen and if transcriptome and proteome are quantified in the same conditions (relevant if one is directly to compare both networks). Provide also details about the number of link/nodes of each subnetwork and corresponding subgraph. Since transcriptomic data are provided usually in FPKM and proteomic in counts (sum of normalized intensities of each ion channel), are data further normalized to facilitate their comparison?

RESPONSE: The number of samples in each dataset has now been specified in the Methods, as well as the matching between CPTAC and TCGA samples. Since each dataset has been used independently for network construction, we did not employ any additional normalization to directly integrate the different data types.

===

3.- PPI networks:

a.- Since they are going to compare PPIs about different 'contexts', a brief explanation about the tissue origin and peculiarities of the three cell lines investigated is in order.

RESPONSE: We have added some information about the tissues of origin of the three cell lines.

====

3. b - Please provide details about number of proteins/interactions in the contrast subgraphs obtained from the comparisons of the three cell lines. Since these subgraphs are going to be compared to RNA expression data from a different dataset, please specify if these data are obtained from the same cell lines. Why PPI data are compared only to upregulated genes? (and not to up-down regulated). Also, concerning the criterion for 'upregulation' (logFC>1), is this log base 2?. How do they quantify the overlap between proteins in PPI and upregulated genes? They just state that 'did indeed significantly overlap the corresponding up-regulated genes'. How much is the overlap and what does 'significantly' mean?

RESPONSE: We have specified the size range of the contrast subgraphs obtained in the Results section. The RNA expression data are indeed derived from the same cell lines (these are very widely used cell lines for which many different omics datasets have been made available by many labs and consortia). The significance of the overlap is established using the Fisher exact test, as we have now specified in the Results, where we also quote the overlap size and Fisher P-value for some examples. The reason for considering up-regulated genes is the following: When comparing two PPIs (say HEK293T and HUVEC) we produce two contrast subgraphs, one containing protein pairs which specifically interact in HEK293T and one containing the HUVEC-specific interactions. Genes that are up-regulated in HEK293T are expected to appear in the former, and those up-regulated in HUVEC in the latter. So each contrast subgraph is compared to the list of differentially expressed genes in which it is expected to be enriched. We have clarified this point in the Results. The logFC is indeed in base 2, and we have specified this in the Methods.

=====

3. c -Discussion of the results shown in Figure 4 is not clear to me. First, the authors state 'We thus analyzed in more depth the first contrast subgraphs obtained from the comparison of the HEK293T PPI network with those obtained from the other two cell lines'. Does this mean that they analyze four subgraphs (2 for HEK vs. HUVEC and 2 for HEK vs. Jurkat?. When they say that the 'top contrasts subgraphs were identical', do they mean that the four subgraphs contained exactly the same nodes?. Also, in main text Figure 4 seems to contain the subnetwork of these subgraphs with only the nodes annotated as 'ribosome biogenesis' and 'signal transduction through p53', and the links would be the PPIs. But in the caption to Figure 4 they state that 'green edges join proteins involved in the two biological processes' (probably a subset of the PPIs). Please clarify. Why do they give only the comparison between HEK and HUVEC, and not between HEK and Jurkat if the same nodes are present?

RESPONSE: We have clarified the procedure in the Results section. We analyze only two contrast subgraphs, those characterized by higher edge density in HEK293T compared to JURKAT (HUVEC). This is because the opposite contrast subgraphs (e.g. higher density in JURKAT compared with HEK293T) are “polluted” by transcriptionally upregulated genes, as discussed above. Also the caption has been clarified. Indeed these are subnetworks of the two PPIs limited to proteins annotated to one or both of the two functions. We chose to show the comparison with HUVEC as an example, but the comparison with JURKAT would be identical, due to the fact that all the interactions between these proteins that are not HEK293T-specific are shared among all three cell lines according to the data of Ref. [16].

====

Interpretation of results:

1.- Coexpression networks in two cancer subtypes: they find that the subgraph with the stronger connections in the basal subtype is enriched in 'immune response' and the subgraph denser in the luminal subtype is enriched in categories related to microenvironment regulation. If they identify clearly enriched genes they should discuss in more depth their known roles in connection to these two functions in their biological context. This would enrich and support their findings. It is tempting to speculate that, since the basal type is less aggressive, cancer cells are challenged by the immune system of the organism but, once they developed mechanisms to evade the immune system (becoming more aggressive as in the luminal subtype) they are committed to manipulate their microenvironment to proliferate. Are there any evidences for this in these subtypes of cells?

RESPONSE: According to the reviewer’s advice, we have expanded the discussion on the biological interpretation of the identified differential subgraphs and enriched biological categories. Indeed, most aggressive subtypes such as the basal-like are characterized by higher T lymphocytes infiltration and higher extracellular matrix stiffening (doi: 10.1039/c5ib00040h), which fits with the biological function of genes in the identified differential subgraphs (e.g. metalloproteinases contributing to extracellular matrix loosening in luminal-A).

====

2. - Comparison of transcriptomic and proteomic networks: From their analyses in Figure 3 they claim in the Discussion that 'adaptive immune system genes are more connected at the transcriptional level, while innate immune systems are more connected at the proteomic level'. This is a rather vague statement based on the functional enrichment analysis. First, they should identify and discuss in more detail the genes/proteins responsible for this enrichment, to see if their documented function supports their speculations (and since the data they use are from breast cancer, I don't know how general could be this observation of if it is specific of this type of tumor). Moreover, caution should be exerted when interpreting these coexpression networks: the most connected transcripts are not necessarily those who are being simultaneously translated. Also, since apparently the network is not signed the abundance of connected transcripts may be anticorrelated. Finally, Figure 3 is not
clear: which panel corresponds to the transcriptomic subgraph and which one to the proteomic one? This should be specified in the caption or with titles in the panel.

RESPONSE: According to the reviewer's suggestion, we have now significantly expanded the biological discussion. In particular, genes coding for metalloproteinases, complement cascade proteins and regulators of the complement system have now been included as specific examples in a wider discussion of the biological interpretations of our computational results, comparing the biological implications with previous knowledge on differences in the microenvironment composition across breast cancer subtypes and in the regulatory mechanisms of humoral and adaptive immunity.
We apologize for the captions' lack of clarity. We have now added titles in the panels.

====

Minor comments:

- The distinction between 'heterogeneous' and 'homogeneous' networks in the Introduction is a bit confusing, as they classify mRNA and protein coexpression networks as 'heterogeneous'. Why is that? Is that because they are built from many different samples/individuals or time course data?.

RESPONSE: "Homogeneous" and "heterogeneous" refer to the experimental assays used to produce the data. So when comparing RNA-based coexpression networks (section "Coexpression networks in two subtypes of breast cancer") or protein-protein interaction networks (section "Protein interaction networks in human cell lines") we are comparing homogeneous networks, while in section "Protein vs mRNA coexpression in breast cancer" we are comparing heterogeneous networks (one derived from transcriptomics and one from proteomics). We have explicitly classified the three examples as homogeneous vs heterogeneous in the revised version.

====

Although I have nothing against how the authors display differences between the first contrast subgraphs in panels A-B of Figures 1 and 2, it may be more eye-catching to display these differences as usual boxplots or violin plots, with perhaps the test for significant differences between the means of both degree distributions.

RESPONSE: The figure as it stands corresponds to juxtaposed halved violin plots: The complete violin plots would not add any more information, so also for the sake of space we would like to leave them as they are. We have added P-values from the Mann-Whitney U test.

====

Reviewer 3
The authors apply the contrast subgraph analysis to three different omics data sets - overall these analysis are not very detailed and do not yield surprising results but they provide a nice illustration of the potential usefulnes of the contrast subgraph analysis in the context of omics data. To my opinion this is really where the merit of the paper is: to promote and make accessible the method to a wider audience of researchers in the field of bioinformatics/molecular biology.

One of the downsides of the presented approach is that it does not provide any measures of confidence in the results - while there is a parameter >alpha< that allows some tuning, little information is given on how to choose a suitable value for this parameter (which obviously depends on the data).

RESPONSE: Regarding the tunable parameter alpha, this was used in the algorithm presented in the original Lanciano et al. paper, but since this algorithm did not easily scale to the network sizes typical of omics data, we have used instead “Algorithm 1” described in the Methods section, which actually does not contain any tunable parameters.

We agree with the reviewer that a potential weakness of the method is the lack of a quantitative measure of confidence in the results, and we have underlined such limitation in the Discussion. Indeed this limitation is shared by community detection algorithms, of which ours can be considered, as discussed in this revised version, as a “supervised version” where we maximize a function related to the difference in modularity, rather than modularity itself.

However we believe that the robustness of the results with respect to the use of different datasets (TCGA vs METABRIC) and different association metrics (correlation vs proportionality, the latter added in this revised version, see response to the next comment) allow us, indirectly, to be quite confident in the biological validity of the results, although we recognize that such extrinsic controls are not always available in practice

=====

Another issue that might come a little too short is how to derive graph representations from experimental omics data in the first place. Usually these methods do not yield yes/no answers, but rather we obtain a matrix of pairwise measurements (e.g. correlation of coexpression) and to obtain a graph a threshold on these numbers is applied to obtain an edge or not. Various methods have been proposed to choose thresholds, but in the end, moving from a full matrix to graph representation means loosing some information - it would be interesting to see a deeper analysis on how much this thresholding influences the outcomes of the proposed method - this question is obviously linked to obtaining some confidence information on the results.

RESPONSE: The coexpression networks are built as in WGCNA (ref. [10]) and are thus complete and do not require thresholding (although soft thresholding is provided, as in WGCNA, by elevating (1+r) to a rather high power - we use the default WGCNA power of 12).

In this revised version we also added an analysis of the subtype-specific breast cancer coexpression networks based on proportionality instead of correlation, showing that the contrast subgraphs derived with the two methods largely overlap. Thus, while it is certainly true that the construction of coexpression networks from transcriptome data involves many arbitrary choices, we find it reassuring that our results do not seem to depend strongly on such choices. The robustness of the results with respect to both network construction procedure and choice of dataset (e.g. TCGA or METABRIC) allow us to be confident about the biological meaning of these contrast subgraphs also in the absence of a quantitative measure of confidence.

====

-figure captions esp 1-3 - please provide more information in the figure captions to make the figures "readable" on their own without a need for the reader to refer back to the text; figure captions for Fig 1-3 are almost identical, yet very different data is shown - a clear indication that important information is missing in the figure caption - such as what is the underlying data?
Please explain all terms used in the figure in its caption: here what is "GeneRatio"? Figs A/B what is the x-axis showing for the violin plots?

RESPONSE: We apologize for the lack of clarity. We have now added titles and specifics of the data represented.

=====

-figure 3c and para on Protein vs mRNA coexpression (p2-5) - are the differences really that striking - in 3C, the box plots do not look that different, super low p-values are probably due to very large number of data points, but not sure it is really that meaningful here (effect size?)

RESPONSE: We have included a measure of the effect size (Cohen’s d = 0.52).

=====

-figure 4 is too small, nodes are barely visible, colours cannot be distinguished

RESPONSE: We resized the figure, making it larger.

====

-algorithm 1 and description in text - I would probably move the description of the algorithm from the text to a "figure caption" for the algorithm box, to make it easier for the reader to find the definitions of the terms

RESPONSE: We added a detailed description of the algorithm just below the pseudo-code.

Close
