# Supplementary material for: Contrast subgraphs allow comparing homogeneous and heterogeneous networks derived from omics data
Source: Gigascience. 2023 Feb 28;12:giad010. doi: 10.1093/gigascience/giad010 (PMC9972522; doi:10.1093/gigascience/giad010)
Supplement: giad010_Response_to_Reviewer_Comments_Revision_1 [file giad010_response_to_reviewer_comments_revision_1.pdf]

Author's Response To Reviewer Comments

Close

We have made all the changes required. Please note that the ORCID ids are included as comments in the "main.tex" file among the latex source files.

Close
