# Supplementary material for: Contrast subgraphs allow comparing homogeneous and heterogeneous networks derived from omics data
Source: Gigascience. 2023 Feb 28;12:giad010. doi: 10.1093/gigascience/giad010 (PMC9972522; doi:10.1093/gigascience/giad010)
Supplement: giad010_Reviewer_1_Report_Original_Submission — De-Shuang Huang -- 9/12/2022 Reviewed [file giad010_reviewer_1_report_original_submission.pdf]

Reviewer Report

Title: Contrast Subgraphs Allow Comparing Homogeneous and Heterogeneous Networks Derived from Omics Data

Version: Original Submission **Date:** 9/12/2022

Reviewer name: De-Shuang Huang

Reviewer Comments to Author:

The authors proposed an algorithm based on contrasting subgraphs to characterize the biological networks, so as to analyze the specificity and conservation between different samples. It is interesting and I think there are some problems that need to be clarified.1, Sub-graphs are generated by dividing the whole graph in a certain way, and the similarity and difference of the samples are described by the comparison between the sub-graphs. The authors should discuss the advantages of the proposed approach in a non-heuristically way compared with the previous methods. Besides that, I wonder why subgraphs need to be non-overlapping.2, For TCGA or other databases, I think the authors should state the details of the samples, such as the number of samples, sequencing technology, batch effects, etc. In addition, the authors should describe the relationship between the subgraphs and GO modules to explain the results and draw some biological conclusions.3, The authors performed a similar analysis on protein networks and compared the results with RNA-seq, and get some conclusions. I'm a little confused whether the GO enrichment analysis of proteomics is to map the protein ID to the gene ID. If so, the authors can easily combine transcript co-expression and protein co-expression networks through ID-to-ID mapping, and I look forward to the results of such an analysis.4, I would like to know how the proposed method handles heterogeneous graphs by treating heterogeneous graphs as Homogeneous graph to generate subgraphs? I didn't figure out which dataset is the heterogeneous graph scenario.5, In addition to the elaboration of results such as degree and density differences between subgraphs, I would like to see the relationships between these results and the biological problems.6, Authors may consider citing the following articles on networks in molecular biologyBarabasi A L, Oltvai Z N. Network biology: understanding the cell's functional organization[J]. Nature reviews genetics, 2004, 5(2): 101-113.Zhang, Q., He, Y., Wang, S., Chen, Z., Guo, Z., Cui, Z., ... & Huang, D. S. (2022). Base-resolution prediction of transcription factor binding signals by a deep learning framework[J]. PLoS computational biology, 2022, 18(3): e1009941.Hu J X, Thomas C E, Brunak S. Network biology concepts in complex disease comorbidities[J]. Nature Reviews Genetics, 2016, 17(10): 615-629.Z.-H. Guo, Z.-H. You, Y.-B. Wang, D.-S. Huang, H.-C. Yi, and Z.-H. Chen, "Bioentity2vec: Attribute-and behavior-driven representation for predicting multi-type relationships between bioentities." GigaScience 9.6 (2020): giaa032.Z.-H. Guo, Z.-H. You, D.-S. Huang, H.-C. Yi, K. Zheng, Z.-H. Chen, Y.-B. Wang, MeSHHeading2vec: a new method for representing MeSH headings as vectors based on graph embedding algorithm[J]. Briefings in bioinformatics, 2021, 22(2): 2085-2095.

Level of Interest

Please indicate how interesting you found the manuscript: Choose an item.

Quality of Written English

Please indicate the quality of language in the manuscript: Choose an item.

Declaration of Competing Interests

Please complete a declaration of competing interests, considering the following questions:

- Have you in the past five years received reimbursements, fees, funding, or salary from an organisation that may in any way gain or lose financially from the publication of this manuscript, either now or in the future?
- Do you hold any stocks or shares in an organisation that may in any way gain or lose financially from the publication of this manuscript, either now or in the future?
- Do you hold or are you currently applying for any patents relating to the content of the manuscript?
- Have you received reimbursements, fees, funding, or salary from an organization that holds or has applied for patents relating to the content of the manuscript?
- Do you have any other financial competing interests?
- Do you have any non-financial competing interests in relation to this paper?

If you can answer no to all of the above, write 'I declare that I have no competing interests' below. If your reply is yes to any, please give details below.

I declare that I have no competing interests

I agree to the open peer review policy of the journal. I understand that my name will be included on my report to the authors and, if the manuscript is accepted for publication, my named report including any attachments I upload will be posted on the website along with the authors' responses. I agree for my report to be made available under an Open Access Creative Commons CC-BY license (http://creativecommons.org/licenses/by/4.0/). I understand that any comments which I do not wish to be included in my named report can be included as confidential comments to the editors, which will not be published.

Choose an item.

To further support our reviewers, we have joined with Publons, where you can gain additional credit to further highlight your hard work (see: https://publons.com/journal/530/gigascience). On publication of this paper, your review will be automatically added to Publons, you can then choose whether or not to claim your Publons credit. I understand this statement.

Yes Choose an item.
