# Supplementary material for: Contrast subgraphs allow comparing homogeneous and heterogeneous networks derived from omics data
Source: Gigascience. 2023 Feb 28;12:giad010. doi: 10.1093/gigascience/giad010 (PMC9972522; doi:10.1093/gigascience/giad010)
Supplement: giad010_Reviewer_2_Report_Original_Submission — Raul Guantes -- 9/19/2022 Reviewed [file giad010_reviewer_2_report_original_submission.pdf]

Reviewer Report

Title: Contrast Subgraphs Allow Comparing Homogeneous and Heterogeneous Networks Derived from Omics Data

Version: Original Submission **Date:** 9/19/2022

Reviewer name: Raul Guantes

Reviewer Comments to Author:

In this manuscript the authors apply the method of contrast subgraphs (developed among others by some of the authors), that identifies salient structural differences between two networks with the same nodes, to several biological co-expression and PPI networks. This method adds to the extensive toolkit of network analyses that have been used in the last two decades to extract useful biological information from omics data. In particular, the authors identify subgraphs containing maximum differences in connectivity between two networks, and basically use functional annotations to assign biological meaning to these differences. Of note, contrast subgraphs is not the only method that provides 'node identity awareness' when comparing networks. For instance, identification of network modules or community partitions are common methods to identify groups of nodes that highlight potentially relevant structural differences between two networks, and have been applied to many biological and other types of networks.I find the manuscript well motivated and clearly written in general, but lacking detailed information on part of the Methods. The discussion connecting their findings on structural differences between networks to potential biological functions is also a bit vague and could be worked out in more detail. I feel that the paper is potentially acceptable in GigaScience after a revision to provide more details on the methods and on their findings. Here are my comments:Methods:1.- Coexpression networks for luminal and basal cancer subtypes:1a.- The authors don't give enough information about the data they are using to build these networks. How many samples/points are they using to calculate correlations? Do they correspond to different patients, expression dynamics after some treatment…? Is there any preprocessing in the data (e.g. differential expression with respect to healthy tissue) or they just take all quantified transcripts and proteins with minimal filtering (they only specified that filter out genes with FPKM < 1 in more than 50 samples in transcriptomic data)? How many nodes and links have the final coexpression networks?.1b.- To determine links between genes/proteins they calculate Spearman rho and transform it to (0.5(1+rho)^12 to give a 'signed' network. But since Spearman correlation ranges between +1 and -1, this transformed quantity lies between 0 and 1, so I don't see the sign. Moreover, why the exponent 12 in the transformation??. Please clarify because I don't know if they are analyzing just weighted networks, unweighted networks or signed networks in the end because somehow they 'keep track' of the sign of rho. They spend some space in Methods discussing the extension of the contrast subgraph method to sign networks, but I don't know if they finally apply it, since coexpression networks built in this way and PPI networks are not signed.1c.- Do they keep all links or use some cutoff in rho by magnitude/significance? Presumably yes, because otherwise the final network would be a clique and unmanageable, but they don't give any info on that. Again, which is the final size (node/links) of the coexpression networks?1d.- As for coexpression

networks based on relative abundance data as those from transcriptomic/proteomic experiments, it is well known that correlations may be misleading due to the possible large number of spurious correlations (see for instance Lovell at al., PLoS Computational Biology 11(3) (2015) e1004075). The use of correlations requires some justification, and at least to acknowledge the potential pitfalls of this measure.1e.- How many nodes/links are in the first contrast subgraphs shown in Figures 1-2? Is the degree calculated within the whole network or just within the extracted subgraph?1f.- Page 4, last paragraph before 'Protein vs mRNA coexpression in breast cancer' section: 'the results obtained with the two independent breast cancer cohorts show good agreement, with the top differential subgraphs significantly overlapping for both the basal-like and the luminal-A subtypes (Fisher test p < 2.2 Â· 10-16)'. I guess the overlapping is in terms of functional annotations, how is this overlapping and the corresponding statistical test calculated?.2.- Protein versus mRNA coexpression:2a.- Please provide again information about the number of samples, how the 'subset of breast cancer patients included in the TCGA' is chosen and if transcriptome and proteome are quantified in the same conditions (relevant if one is directly to compare both networks). Provide also details about the number of link/nodes of each subnetwork and corresponding subgraph. Since transcriptomic data are provided usually in FPKM and proteomic in counts (sum of normalized intensities of each ion channel), are data further normalized to facilitate their comparison?3.- PPI networks:3a.- Since they are going to compare PPIs about different 'contexts', a brief explanation about the tissue origin and peculiarities of the three cell lines investigated is in order.3b.- Please provide details about number of proteins/interactions in the contrast subgraphs obtained from the comparisons of the three cell lines. Since these subgraphs are going to be compared to RNA expression data from a different dataset, please specify if these data are obtained from the same cell lines. Why PPI data are compared only to upregulated genes? (and not to up-down regulated). Also, concerning the criterion for 'upregulation' (logFC>1), is this log base 2?. How do they quantify the overlap between proteins in PPI and upregulated genes? They just state that 'did indeed significantly overlap the corresponding up-regulated genes'. How much is the overlap and what does 'significantly' mean?3c.- Discussion of the results shown in Figure 4 is not clear to me. First, the authors state 'We thus analyzed in more depth the first contrast subgraphs obtained from the comparison of the HEK293T PPI network with those obtained from the other two cell lines'. Does this mean that they analyze four subgraphs (2 for HEK vs. HUVEC and 2 for HEK vs. Jurkat?. When they say that the 'top contrasts subgraphs were identical', do they mean that the four subgraphs contained exactly the same nodes?. Also, in main text Figure 4 seems to contain the subnetwork of these subgraphs with only the nodes annotated as 'ribosome biogenesis' and 'signal transduction through p53', and the links would be the PPIs. But in the caption to Figure 4 they state that 'green edges join proteins involved in the two biological processes' (probably a subset of the PPIs). Please clarify. Why do they give only the comparison between HEK and HUVEC, and not between HEK and Jurkat if the same nodes are present?Interpretation of results:1.- Coexpression networks in two cancer subtypes: they find that the subgraph with the stronger connections in the basal subtype is enriched in 'immune response' and the subgraph denser in the luminal subtype is enriched in categories related to microenvironment regulation. If they identify clearly enriched genes they should discuss in more depth their known roles in connection to these two functions in their biological context. This would enrich and support their findings. It is tempting to speculate that, since the basal type is less aggressive, cancer cells are challenged by the immune system of the organism but, once they developed mechanisms to evade the

immune system (becoming more aggressive as in the luminal subtype) they are committed to manipulate their microenvironment to proliferate. Are there any evidences for this in these subtypes of cells?2.- Comparison of transcriptomic and proteomic networks: From their analyses in Figure 3 they claim in the Discussion that 'adaptive immune system genes are more connected at the transcriptional level, while innate immune systems are more connected at the proteomic level'. This is a rather vague statement based on the functional enrichment analysis. First, they should identify and discuss in more detail the genes/proteins responsible for this enrichment, to see if their documented function supports their speculations (and since the data they use are from breast cancer, I don't know how general could be this observation of if it is specific of this type of tumor). Moreover, caution should be exerted when interpreting these coexpression networks: the most connected transcripts are not necessarily those who are being simultaneously translated. Also, since apparently the network is not signed the abundance of connected transcripts may be anticorrelated. Finally, Figure 3 is not clear: which panel corresponds to the transcriptomic subgraph and which one to the proteomic one? This should be specified in the caption or with titles in the panel.Minor comments:- The distinction between 'heterogeneous' and 'homogeneous' networks in the Introduction is a bit confusing, as they classify mRNA and protein coexpression networks as 'heterogeneous'. Why is that? Is that because they are built from many different samples/individuals or time course data?.- Although I have nothing against how the authors display differences between the first contrast subgraphs in panels A-B of Figures 1 and 2, it may be more eye-catching to display these differences as usual boxplots or violin plots, with perhaps the test for significant differences between the means of both degree distributions.

Level of Interest

Please indicate how interesting you found the manuscript: Choose an item.

Quality of Written English

Please indicate the quality of language in the manuscript: Choose an item.

Declaration of Competing Interests

Please complete a declaration of competing interests, considering the following questions:

- Have you in the past five years received reimbursements, fees, funding, or salary from an organisation that may in any way gain or lose financially from the publication of this manuscript, either now or in the future?
- Do you hold any stocks or shares in an organisation that may in any way gain or lose financially from the publication of this manuscript, either now or in the future?
- Do you hold or are you currently applying for any patents relating to the content of the manuscript?
- Have you received reimbursements, fees, funding, or salary from an organization that holds or has applied for patents relating to the content of the manuscript?

- Do you have any other financial competing interests?
- Do you have any non-financial competing interests in relation to this paper?

If you can answer no to all of the above, write 'I declare that I have no competing interests' below. If your reply is yes to any, please give details below.

I declare that I have no competing interests

I agree to the open peer review policy of the journal. I understand that my name will be included on my report to the authors and, if the manuscript is accepted for publication, my named report including any attachments I upload will be posted on the website along with the authors' responses. I agree for my report to be made available under an Open Access Creative Commons CC-BY license (http://creativecommons.org/licenses/by/4.0/). I understand that any comments which I do not wish to be included in my named report can be included as confidential comments to the editors, which will not be published.

Choose an item.

To further support our reviewers, we have joined with Publons, where you can gain additional credit to further highlight your hard work (see: https://publons.com/journal/530/gigascience). On publication of this paper, your review will be automatically added to Publons, you can then choose whether or not to claim your Publons credit. I understand this statement.

Yes Choose an item.
