# Supplementary material for: Contrast subgraphs allow comparing homogeneous and heterogeneous networks derived from omics data
Source: Gigascience. 2023 Feb 28;12:giad010. doi: 10.1093/gigascience/giad010 (PMC9972522; doi:10.1093/gigascience/giad010)
Supplement: giad010_Reviewer_3_Report_Original_Submission — Thomas Schlitt -- 9/23/2022 Reviewed [file giad010_reviewer_3_report_original_submission.pdf]

Reviewer Report

Title: Contrast Subgraphs Allow Comparing Homogeneous and Heterogeneous Networks Derived from Omics Data

Version: Original Submission **Date:** 9/23/2022

Reviewer name: Thomas Schlitt

Reviewer Comments to Author:

The manuscript "contrast subgraphs allow comparing homogeneous and hetereogeneous networks derived from omics data" introduces and illustrates the application of contrast subgraph analysis to gene expression, protein expression and protein-protein interaction data. The method can be applied to weighted networks. The authors give a good description of the method and the context of other available methods.The authors apply the contrast subgraph analysis to three different omics data sets - overall these analysis are not very detailed and do not yield surprising results but they provide a nice illustration of the potential usefulnes of the contrast subgraph analysis in the context of omics data. To my opinion this is really where the merit of the paper is: to promote and make accessible the method to a wider audience of researchers in the field of bioinformatics/molecular biology. The authors have also applied their method to brain imaging derived networks, but that work is not part of this publication.The contrast subgraph analysis is particularly interesting, for data that is collected under different conditions but for the same set of nodes (i.e. genes, proteins, ...), i.e. where the nodes present do not change (much), but their interaction strengths differes between conditions. It remains to be seen where this method can deliver unique value that is not achievable by other means, but the approach is very intuitive. Its rationale can be readily understood, reducing the temptation to use it as a "black box" without critically questioning the results as might be the case for more complex methods. One of the downsides of the presented approach is that it does not provide any measures of confidence in the results - while there is a parameter >alpha< that allows some tuning, little information is given on how to choose a suitable value for this parameter (which obviously depends on the data). Another issue that might come a little too short is how to derive graph representations from experimental omics data in the first place. Usually these methods do not yield yes/no answers, but rather we obtain a matrix of pairwise measurements (e.g. correlation of coexpression) and to obtain a graph a threshold on these numbers is applied to obtain an edge or not. Various methods have been proposed to choose thresholds, but in the end, moving from a full matrix to graph representation means loosing some information - it would be interesting to see a deeper analysis on how much this thresholding influences the outcomes of the proposed method - this question is obviously linked to obtaining some confidence information on the results.Overall, the method described here is very interesting, it shares downsides with other graph based methods (thresholding), the biological examples given are brief, but illustrative for the use of the method, the manuscript is well readable. The manuscripts stimulates to add this method to your own toolbox and to apply it to interesting data sets to see if it yields results that were not obvious from other approaches.Minor comments:-figure captions esp 1-3 - please provide more information in the figure captions to make the figures "readable" on their own without a need for the

reader to refer back to the text; figure captions for Fig 1-3 are almost identical, yet very different data is shown - a clear indication that important information is missing in the figure caption - such as what is the underlying data?Please explain all terms used in the figure in its caption: here what is "GeneRatio"? Figs A/B what is the x-axis showing for the violin plots?-figure 3c and para on Protein vs mRNA coexpression (p2-5) - are the differences really that striking - in 3C, the box plots do not look that different, super low p-values are probably due to very large number of data points, but not sure it is really that meaningful here (effect size?)-figure 4 is too small, nodes are barely visible, colours cannot be distinguished-algorithm 1 and description in text - I would probably move the description of the algorithm from the text to a "figure caption" for the algorithm box, to make it easier for the reader to find the definitions of the terms

Level of Interest

Please indicate how interesting you found the manuscript: Choose an item.

Quality of Written English

Please indicate the quality of language in the manuscript: Choose an item.

Declaration of Competing Interests

Please complete a declaration of competing interests, considering the following questions:

- Have you in the past five years received reimbursements, fees, funding, or salary from an organisation that may in any way gain or lose financially from the publication of this manuscript, either now or in the future?
- Do you hold any stocks or shares in an organisation that may in any way gain or lose financially from the publication of this manuscript, either now or in the future?
- Do you hold or are you currently applying for any patents relating to the content of the manuscript?
- Have you received reimbursements, fees, funding, or salary from an organization that holds or has applied for patents relating to the content of the manuscript?
- Do you have any other financial competing interests?
- Do you have any non-financial competing interests in relation to this paper?

If you can answer no to all of the above, write 'I declare that I have no competing interests' below. If your reply is yes to any, please give details below.

I declare that I have no competing interests

I agree to the open peer review policy of the journal. I understand that my name will be included on my report to the authors and, if the manuscript is accepted for publication, my named report including any

attachments I upload will be posted on the website along with the authors' responses. I agree for my report to be made available under an Open Access Creative Commons CC-BY license (http://creativecommons.org/licenses/by/4.0/). I understand that any comments which I do not wish to be included in my named report can be included as confidential comments to the editors, which will not be published.

Choose an item.

To further support our reviewers, we have joined with Publons, where you can gain additional credit to further highlight your hard work (see: https://publons.com/journal/530/gigascience). On publication of this paper, your review will be automatically added to Publons, you can then choose whether or not to claim your Publons credit. I understand this statement.

Yes Choose an item.
